# Synthesis and Properties of BODIPY Appended Tetraphenylethylene Scaffolds as Photoactive Arrays

**DOI:** 10.1002/ejoc.202100629

**Published:** 2021-08-04

**Authors:** Harry C. Sample, Ganapathi Emandi, Brendan Twamley, Nitika Grover, Bhavya Khurana, Vincent Sol, Mathias O. Senge

**Affiliations:** ^1^ School of Chemistry Trinity College Dublin The University of Dublin Trinity Biomedical Sciences Institute 152–160 Pearse Street Dublin 2 Ireland; ^2^ School of Chemistry Trinity College Dublin The University of Dublin Dublin 2 Ireland; ^3^ Université de Limoges Laboratoire PEIRENE, EA 7500 8700 Limoges France; ^4^ Institute for Advanced Study (TUM-IAS) Technical University of Munich Focus Group – Molecular and Interfacial Engineering of Organic Nanosystems Lichtenbergstrasse 2a 85748 München Garchig Germany

**Keywords:** Aggregation induced emission, BODIPY, Photosensitizer, Singlet oxygen, Tetraphenylethylene

## Abstract

Tetraphenylethylene (TPE) and its derivatives exhibit excellent aggregation‐induced emission (AIE) properties. The TPE unit is easily accessible, and many functional groups can be introduced in a facile manner to yield effective luminescent materials in both solution and the solid‐state. It is because of this, several TPE‐based compounds have been developed and applied in many areas, such as OLEDs and chemical sensors. Boron dipyrromethenes (BODIPYs) are a class of pyrrolic fluorophore of great interest with myriad application in both material science and biomedical applications. Through the combination of Pd‐catalyzed cross‐coupling reactions and traditional dipyrromethene chemistry, we present the syntheses of novel tetra‐BODIPY‐appended TPE derivatives with different distances between the TPE and BODIPY cores. The TPE‐BODIPY arrays **6** and **9** show vastly differing AIE properties in THF/H_2_O systems, with **9** exhibiting dual‐AIE, along with both conjugates being found to produce singlet oxygen (^1^O_2_). We presume the synthesized BODIPY‐appended TPE scaffolds to be utilized for potential applications in the fields of light‐emitting systems and theranostics.

## Graphical abstract

Herein the synthesis of two novel tetra‐BODIPY tetraphenylethylene (TPE) conjugates is presented. We find differing AIE responses dependent upon the linkage between the TPE and BODIPY cores, as well as differing singlet oxygen generation of the synthesized conjugates.

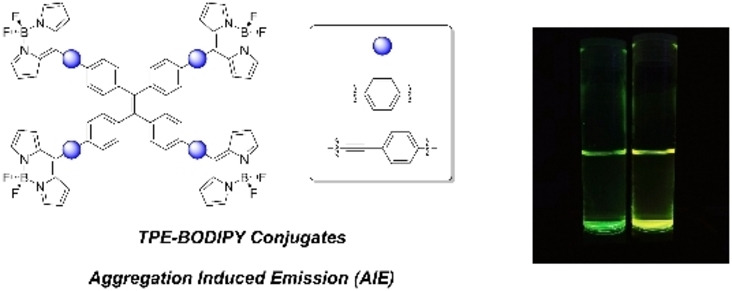

## Introduction

Since the first synthesis of the tetraphenylethylene (TPE) motif in 1907,^[1]^ it has been of considerable interest given its electrochemical and photophysical properties.[^2^, ^3^] Applications of this motif are far reaching and all encompassing; optomechanical switching and storage devices,[^4^, ^5^, ^6^] fluorescent bio‐ and chemo‐sensors,^[7]^ dyes for living cell imaging,^[8]^ components of dye‐sensitized solar cells,^[9]^ and much besides.^[10]^ In 2001, Tang and coworkers,^[11]^ reported a novel class of organic fluorophore which were non‐ or weakly emissive in solution, but upon aggregation or in the solid state these molecules displayed strong fluorescence. This novel phenomenon was coined ‘aggregation induced emission’ (AIE). The TPE motif is of interest given its enhancement of AIE. In the hope of inducing AIE, the TPE motif has been grafted onto a variety of dyes, pyrrolic or otherwise.[^12^, ^13^, ^14^, ^15^, ^16^]

Whilst the use of the TPE core has itself yielded intriguing results in myriad of areas of materials chemistry which has been duly catalogued previously,^[11]^ the incorporation of a dye yields a second response to monitor, i. e., photoluminescence intensity (AIE from the TPE moiety) and electronic absorption (photochemistry from the dye moiety). However usually these systems consist of one TPE and one dye moiety, or one of each in the repeating unit of a polymeric material. E.g., TPE‐hemicyanine conjugates have been utilized in the sensing of homocysteine, cysteine, and glutathione, all molecules which play essential roles in biological processes such as homeostasis and detoxification.^[15]^ TPE‐triphenylamine conjugates have been utilized as the donor part of donor‐acceptor (D‐A) systems in the generation of dye‐sensitized solar cells (DSSCs) and were found to be suitably efficient.^[16]^ Sensing is a major contributor to the uses of TPE; TPE‐carbazole co‐polymers have been found to sense 2,4,6‐trinitrotoluene (TNT),^[17]^ and TPE‐pilar[5]arene conjugates have also been found to selectively sense 4‐amino azobenzene (Oil Yellow B), a frequently used carcinogenic organic dye.^[18]^ TPEs and BODIPYs have been conjugated previously, and Dhokale *et al*. reported that extensive π‐stacking and D‐A type interactions made the resulting conjugates inactive with regards to AIE.^[19]^

**Figure 1:**
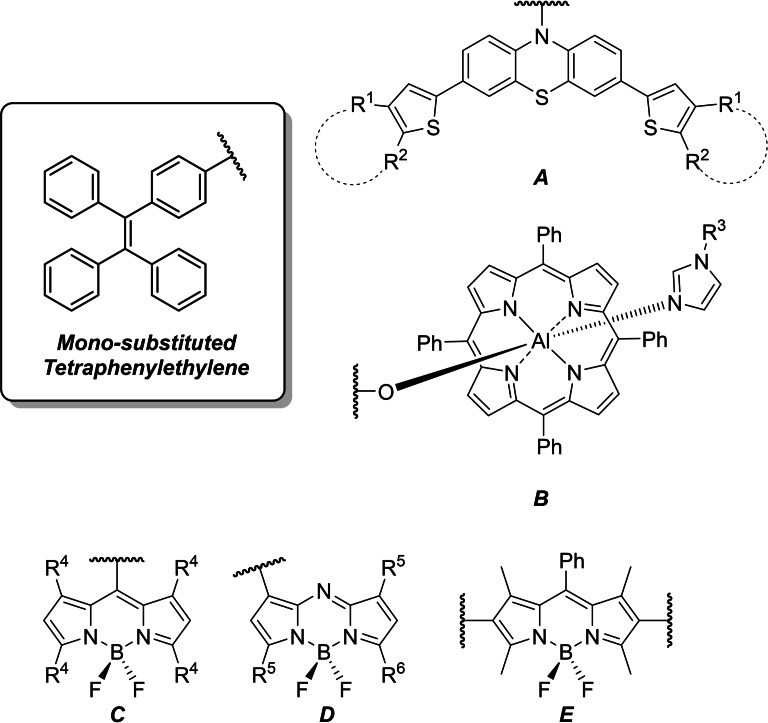
Various dyes onto which the TPE motif has been grafted. A) phenothiazine dyes,^[9]^ R^1^,R^2^=various fused thiophene moieties, B) aluminum porphyrins where R^3^=*p*‐C_6_H_4_‐C_60,_ ^[12]^ and C,D,E) various BODIPYs and aza‐BODIPYs with substitution on all possible positions.[^8^, ^13^, ^14^]

In other previous examples of BODIPY‐TPE conjugates (Figure 1), it is apparent that the most ubiquitous systems utilize mono‐substitution of the fluorophore moiety with a singular TPE unit, either through Pd‐catalyzed cross coupling type reactions (Figure 1, E), utilizing the TPE‐moiety as the meso‐aryl group through standard BODIPY syntheses (Figure 1, D), or otherwise building the fluorophore around the TPE unit (Figure 1, D). Whilst tetra‐substituted TPEs are utilized continually in the fields of metal, and covalent, organic framework (MOF/COF) chemistries – to our knowledge nothing has been explored in the form of a tetra‐dye appended TPE.

Given the propensity for the TPE unit to lend itself of such a wide range of applications and our interest in new BODIPY photonics applications,^[20]^ we present the first tetra‐BODIPY‐appended TPE conjugates. Our approach entailed building the BODIPY onto the TPE through the generation of TPE‐aldehydes through differing Pd‐catalyzed cross‐coupling reactions, and subsequent BODIPY syntheses therefrom. We have evaluated our target compounds regarding their AIE properties, their singlet oxygen (^1^O_2_) production, and discuss their suitability as multi‐photosensitizer arrays. We propose the use of differing arms to link the TPE and BODIPY cores will present altered spectroscopic responses in both solution and aggregated states.

## Results and Discussion

**Figure 2:**
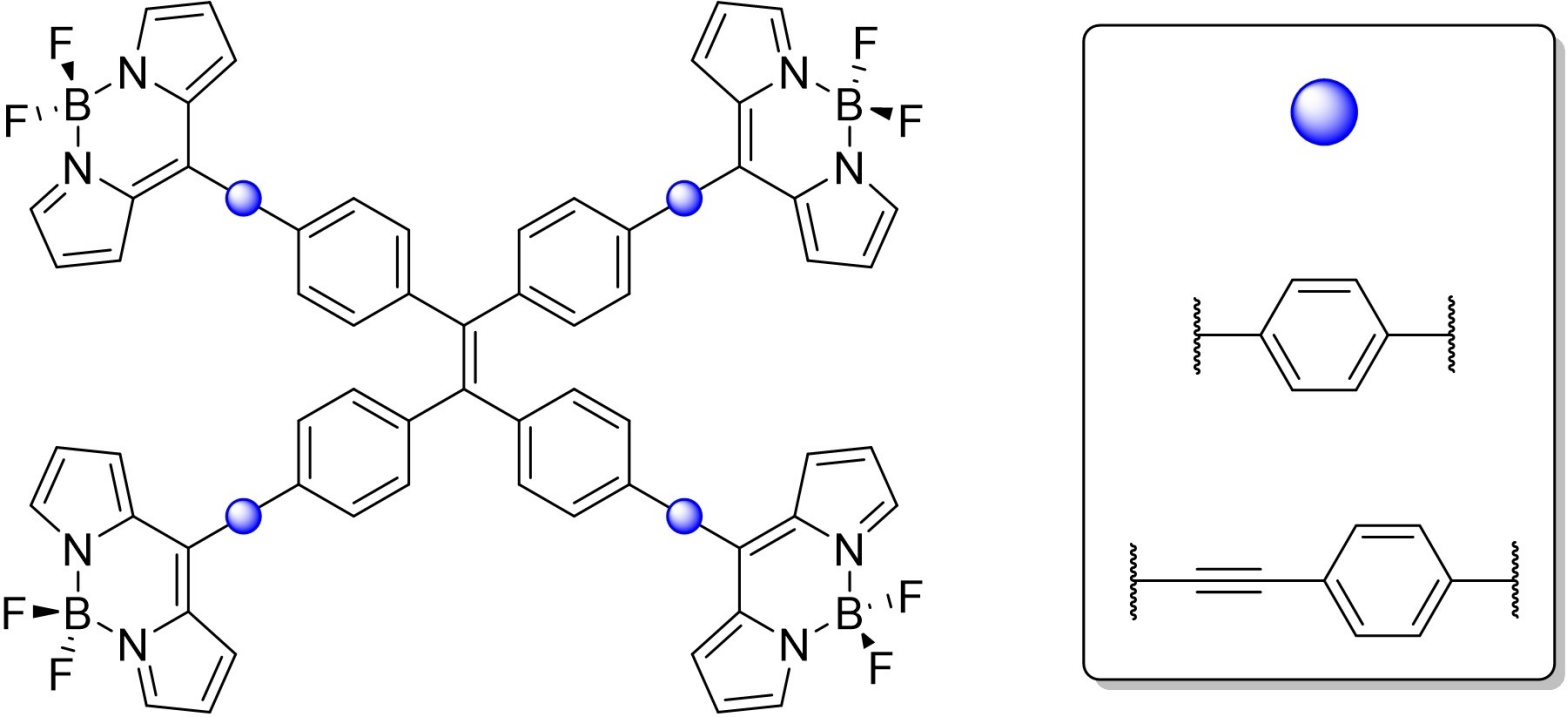
Initial synthetic targets of this study.

**Synthesis** Our initial targets were the structures presented below in Figure 2. In the simplest sense, differing linkages between the TPE core, and four BODIPYs. Through the modification of the linkages between the two cores we envisioned the possibility of differing electronic properties between the two conjugates.

**Scheme 1:**
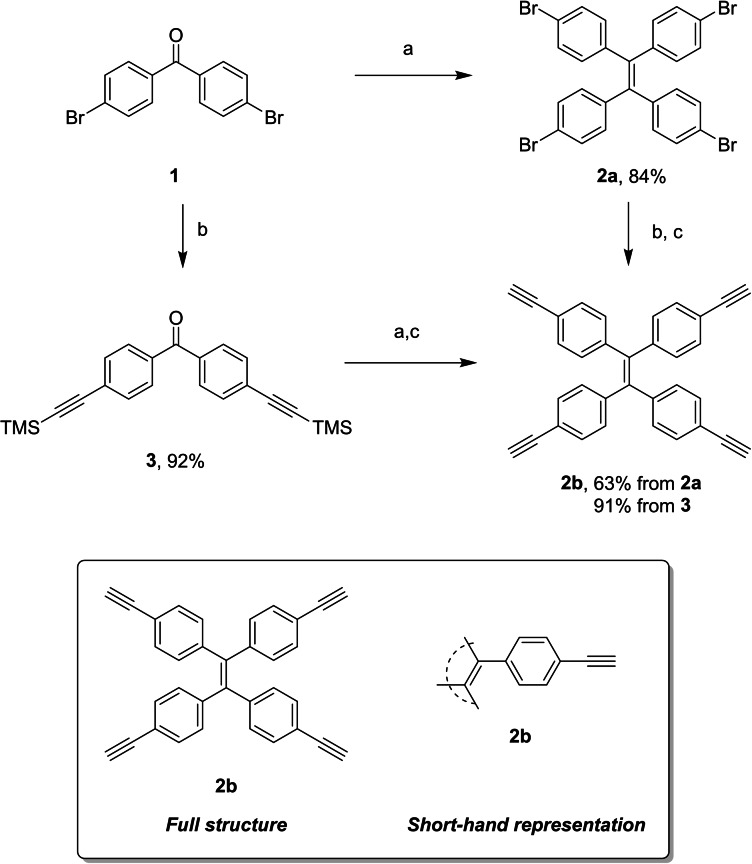
Synthesis of TPE‐Br_4_, **2 a**, and TPP‐(CCH)_4_, **2 b**. Inset: reduced representation of TPE's, with **2 b** used as example. Reagents and conditions: a) Zn, TiCl_4_, THF, 65 °C, 12 h, 84 % for **2 a**, b) TMS‐CCH, CuI (20 mol %), Pd(PPh_3_)_2_Cl_2_ (20 mol %), Et_3_N, THF, 92 % for **3**, c) K_2_CO_3_/THF, 63 % from **2 a** (over two steps), 91 % from **3**.

Synthesis of TPE precursors began from 4,4’‐dibromobenzophenone, **1**, to yield **2 a**, **2 b** and **3** (for X‐ray structure see SI**)** following literature procedures (Scheme 1).^[21]^ Under standard McMurry conditions, TPE‐Br_4_ **2 a** was produced cleanly in 84 % yield. Subsequent Sonogashira coupling with TMS‐acetylene, followed by K_2_CO_3_‐mediated TMS deprotection yielded the tetra‐alkynyl TPE, TPE‐(CCH)_4_ **2 b** in 63 %. Subsequently, through the generation of **2 a** and **2 b**, different routes have been taken to yield tetra‐BODIPY‐TPE arrays where the linkages between the two cores vary both in length and degree of electronic communication.

**Scheme 2:**
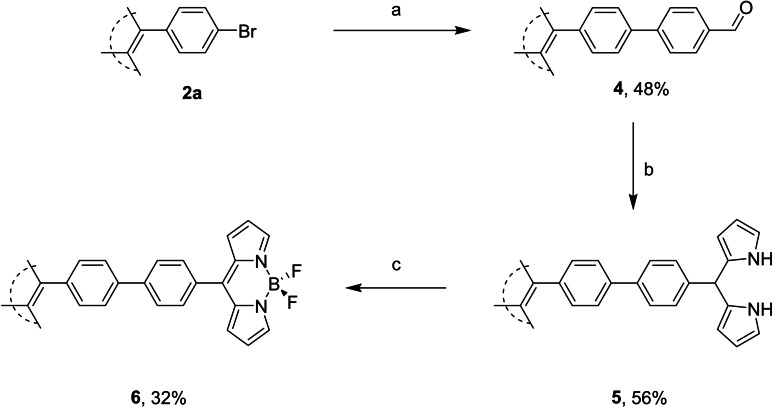
Synthesis of TPE‐*p*‐C_6_H_4_‐CHO, **4**, and subsequent TPE‐*p*‐C_6_H_4_‐BODIPY **6**. Reagents and conditions: a) 4‐formylphenylboronic acid, Cs_2_CO_3_, Pd(PPh_3_)_4_, THF, 70 °C, 24 h, 48 %, b) pyrrole, InCl_3_, CH_2_Cl_2_, r.t., 1 h, 56 % c) DDQ, Et_3_N, BF_3_ ⋅ OEt_2_, CH_2_Cl_2_, r.t., 0.75 h, 32 %.

**Scheme 3:**
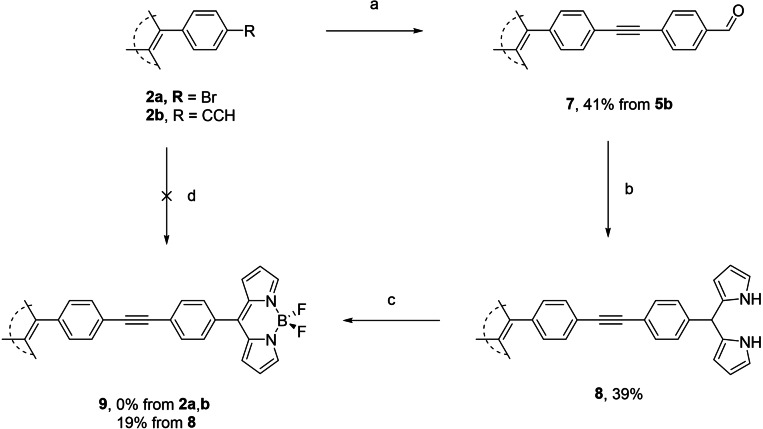
Synthesis of TPE‐CC‐*p*‐C_6_H_4_‐CHO, **7**, and subsequent TPE‐*p*‐C_6_H_4_‐CC‐BODIPY, **9**. Reagents and conditions: a) 4‐iodobenzaldehyde, Pd(PPh_3_)_4_, PPh_3_, CuI, Et_3_N, THF, 41 %; b) pyrrole, InCl_3_, CH_2_Cl_2_, 39 %; c) DDQ, Et_3_N, BF_3_ ⋅ OEt_2_, CH_2_Cl_2_, 19 %; d) 5‐(4‐ethynylphenyl)‐BODIPY (or 5‐(4‐bromophenyl)‐BODIPY), Pd(PPh_3_)_2_Cl_2_ (20 mol %), CuI (20 mol %), PPh_3_, Et_3_N, THF : C_6_H_5_CH_3_, 65 °C, 24 h, 0 %.

**Figure 3:**
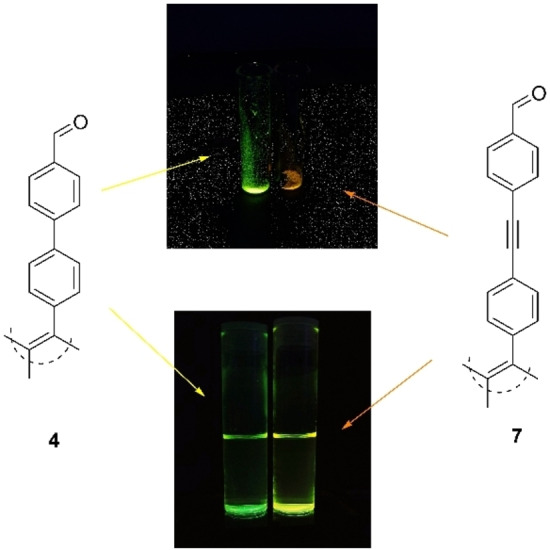
Structures and photographs of aldehydes **4** and **7** being illuminated under UV‐light (360 nm) in the solid state (top) and solution state (THF, bottom).

Treatment of **2 a** with 4‐formylphenylboronic acid under Suzuki conditions yielded TPE‐biphenyl‐aldehyde **4** in 48 % yield (Scheme 2) (for X‐ray structure see SI). With regards to the synthesis of dipyrromethanes (DPMs), there are two standard methods of catalysis; InCl_3_ or trifluoroacetic acid.[^20a^, ^22^] Given the milder conditions with InCl_3_‐mediated catalysis, along with the lesser formation of the respective tripyrranes, we opted for these conditions, and the respective tetra‐dipyrromethane **5** was obtained in 56 % yield. Treatment of **5** in the fashion similar to BF_2_‐insertion for more typical DPMs yielded the tetra‐BODIPY **6** in 32 % yield. Attempts at the utilization of coupling reactions to form **9** were both unsuccessful starting from **2 a** or **2 b** and the respective BODIPY (5‐(4‐bromophenyl)BODIPY or 5‐(4‐ethynylphenyl)BODIPY), in our hands. Thus, akin to displayed previously the DPM was built in a stepwise fashion. Treatment of **2 b** with 4‐iodobenzaldehyde under Sonogashira conditions yielded tetra‐aldehyde **7** in 41 %, and subsequent InCl_3_‐mediated DPM synthesis yielded tetra‐DPM **8** in 39 %. Finally, analogous complexation of BF_2_ gave tetra‐BODIPY **9** in 19 % (Scheme 3). This yield is lowered with respect to previously synthesized *p*‐C_6_H_4_−CCX (X=H, Si(CH_3_)) BODIPYs.^[23]^ However, given that this is a tetra‐BF_2_ insertion, a reduced yield is anticipated. Interestingly, novel aldehyde **7** differed significantly from **4** in the response upon UV illumination in the solid state, varying in both wavelength, and intensity (Figure 3).

**Figure 4:**
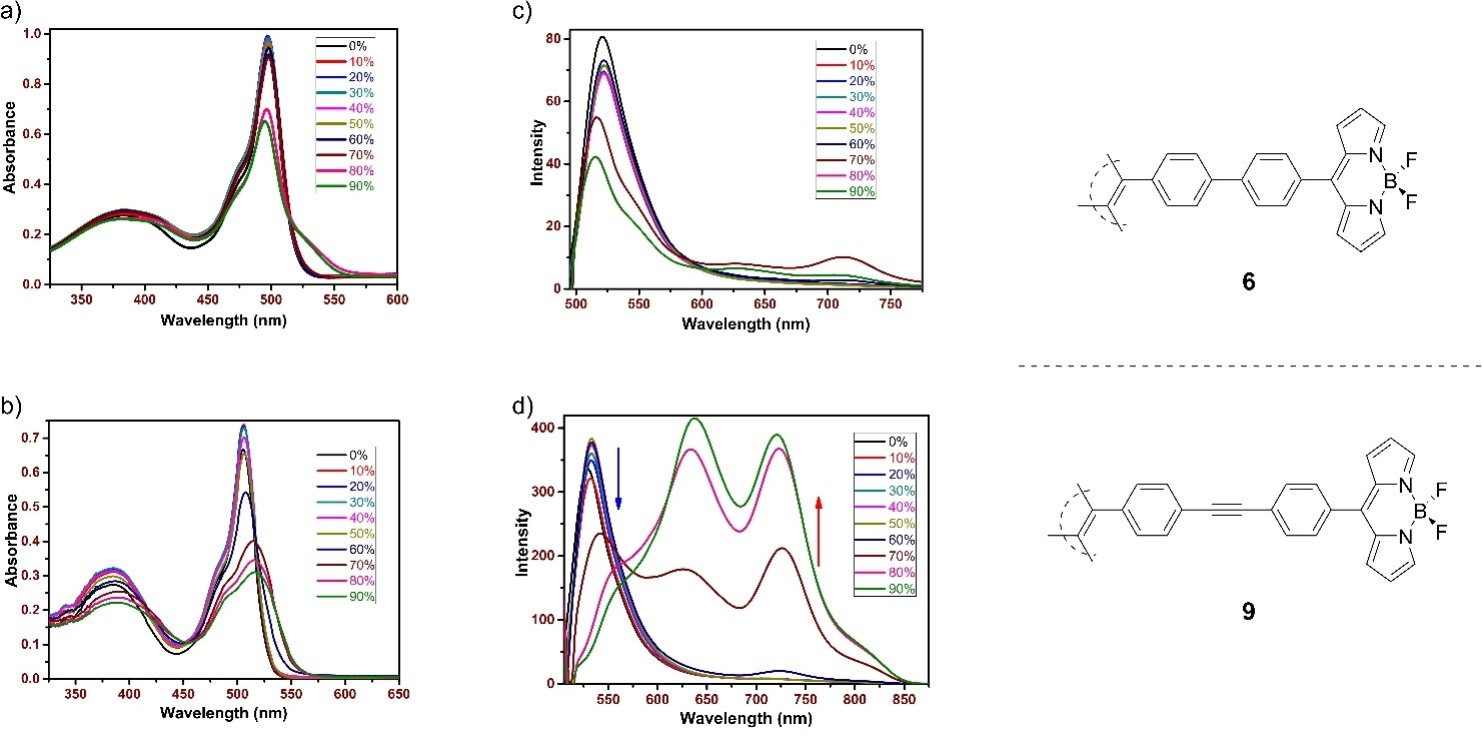
UV‐Vis absorption (a, b), and: emission (c, d) spectra for **6** (a, c) and **9** (b, d) through solvent titrations with mixtures of THF and H_2_O with [H_2_O]=0–90 %. [**6**,**9**]=10 μM.

**Aggregation Induced Emission Analysis**. For analysis of AIE properties, **6** and **9** were considered. Structurally, they differ only in the addition of an ethynyl spacer in **9**. Both were analyzed via UV‐Vis spectroscopy in THF/H_2_O mixtures, with THF as the solvent and H_2_O as the anti‐solvent, with increases of [H_2_O]=10 % between measurements. All analyses were performed with solutions of [**6**, **9**]=10 μM, and spectra are presented in Figure 4.

Typically, BODIPYs absorb in the region of λ_abs_=495–515 nm, dependent upon the solvent used for measurement, and the electronic nature of the substituents along with their positioning around the BODIPY core. The same arguments can be applied to their emission wavelengths which are usually of longer wavelength, i. e. λ_em_>530 nm.^[23g]^ Using these factors, we can see that compound **6** exhibits standard photophysical properties for BODIPYs, despite the TPE core. A decrease in absorbance is observed at 70–80 % H_2_O; however, there is no one particular change in the emission intensity for the same compound in the same solvent mixtures. In contrast, compound **9** does show a stark difference. A decrease in absorbance is observed for **9** at 60 % H_2_O, and the emission intensity was found to increase greatly between 60–80 % H_2_O.

Of particular interest in Figure 4d, is the appearance of two emission bands for **9**, present from [H_2_O] >70 %. These two bands at ca. λ_em_=645, 730 nm could prove to be of particular use with regard to theranostic application of drugs, given the deeper tissue penetration of light through biological tissue with increasing wavelength. In the synthesis of previous TPE‐BODIPY conjugates, Hu *et al*. evaluated how the linkage between TPE and BODIPY cores affected the AIE behavior of the conjugates.^[24]^

**Figure 5:**
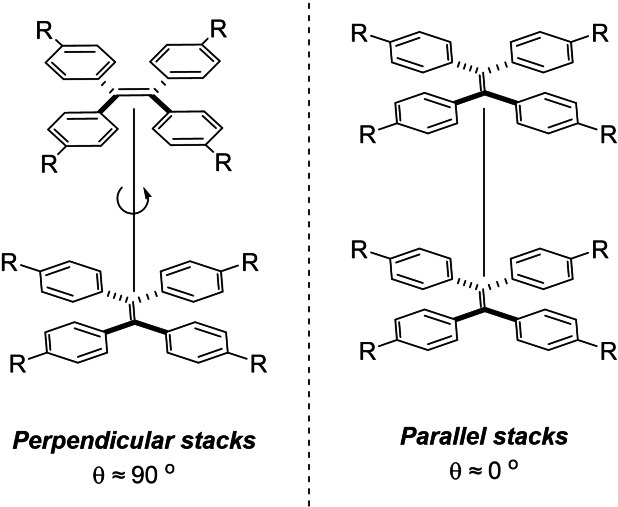
Schematic representation of the differing stackings of TPE‐BODIPY conjugate **9** resulting in aggregation induced dual emission.

For a mono‐BODIPY appended TPE, the spectra presented herein present the same characteristics to those observed previously; in the case of **6** there is a decrease in the locally excited (LE) emission of the BODIPY core, as the water fraction increases, and whilst a new emission does appear around λ_em_=700 nm, the solution is essentially non‐luminescent. For **9** there is again a decrease in the LE emission for the BODIPY core; however, the spectra become dominated by the emissions at ca. λ_em_=645, 730 nm. Given the findings of Hu *et al*. the emission band at λ_em_=645 nm could be a twisted intramolecular charge transfer (TICT) band. However, the second band at λ_em_=730 nm is at very similar intensity to that at λ_em_=645 nm. Such dual aggregation‐induced emission has been observed and examined previously on the diarylethene scaffold.^[25]^ However, the spectra presented of **9** below is indicative of differing π−π stacking modes in the aggregated state (given that any intermolecular vibration is hindered through dense molecular packing, resulting in a dual emission.) Given that the intermolecular packing is so dense, we propose that it is unlikely to be as a result of an end‐to‐face type interaction (i. e. BF_2_ directly interacting with the TPE core), but instead; differing face‐to‐face interactions of the TPE units in perpendicular, or parallel, arrangements. A simplified representation of this is provided in Figure 5. For photographs of the solutions used for these measurements, we refer the readers to Figure S22 and S23.

**Singlet Oxygen (^1^O_2_) Measurements** Given our prior investigations regarding the singlet oxygen generation of BODIPYs,^[20a]^ we have also evaluated compounds **6** and **9** with regards to their singlet oxygen generation. Singlet oxygen (^1^O_2_) is a key species responsible for photochemical and photobiological applications of various photosensitizers.[^20a^, ^20d^] 1,3‐Diphenylisobenzofuran (DPBF) was used as a singlet oxygen trap under generation of *cis*‐dibenzoylbenzene.^[26]^

**Figure 6:**
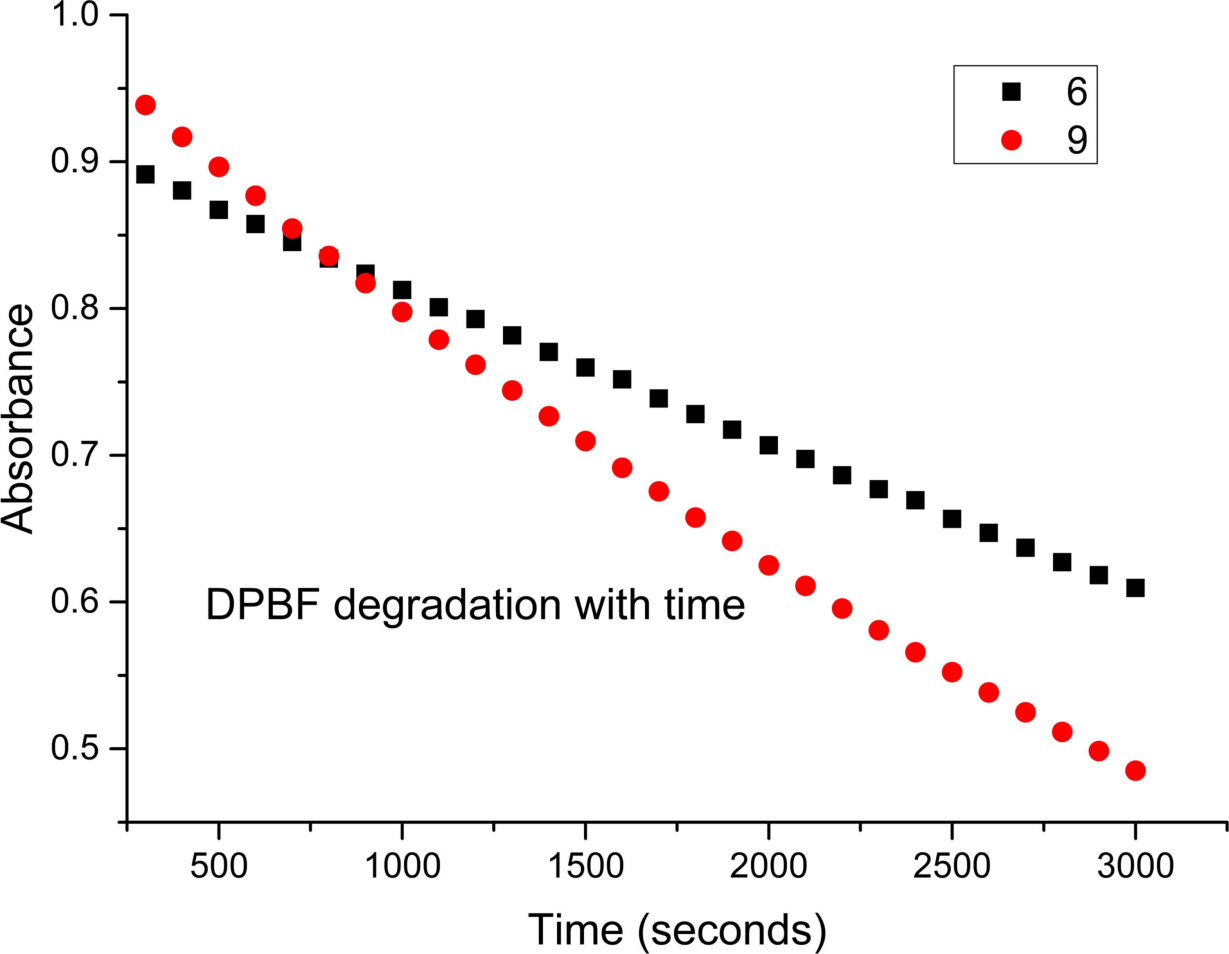
1,3‐diphenylisobenzofuran (DPBF) degradation curves for **6** (black) and **9** (red) in CH_2_Cl_2_ : CH_3_OH, 1 : 1, *v/v*.

The decrease in UV absorbance of DPBF in the presence of both **6** and **9** was measured in CH_2_Cl_2_ : CH_3_OH (1 : 1) with [DPBF]=0.15 M. 5,10,15,20‐tetraphenylporphyrin (H_2_TPP) dissolved in CH_2_Cl_2_ such that [H_2_TPP]=0.15 M was achieved, and subsequently used as a standard (Figure 6). The solutions were irradiated for 1 h and UV‐vis absorption measurements were taken at t=0 seconds and at intervals of 100 seconds after irradiation. Singlet oxygen experiments were repeated twice and Φ_TPE‐BODIPY_ values were observed in a standard deviation range of ±0.05 for both sets of data. The relative quantum yields were calculated with reference to H_2_TPP (Φ_H2TPP_=0.62 in CH_2_Cl_2_).^[27]^

The UV‐Vis absorbance of DPBF was adjusted to *ca*. 1.0 at 417 nm in air‐saturated solvent mixture of CH_2_Cl_2_ : CH_3_OH, then the photosensitizers (**6** or **9**) were added to the cuvette. The slope of the curves of absorbance maxima of DPBF at 410 nm *vs*. irradiation time for each photosensitizer were calculated. The singlet oxygen quantum yield (Φ_TPE‐BODIPY_) for each of these TPE‐BODIPY conjugates with H_2_TPP as a reference was calculated using the following equation 1:^[28]^

(1) $$\Phi {}_{\mathrm{TPE}\text{-}\mathrm{BODIPY}}=\Phi {}_{H2\mathrm{TPP}}\;x\;S{}_{\mathrm{TPE}\text{-}\mathrm{BODIPY}}/S{}_{H2\mathrm{TPP}}$$

where Φ represents quantum yield, S represents the slope, and TPE‐BODIPY and TPP represent TPE substituted BODIPY and 5,10,15,20‐tetraphenylporphyrin, respectively. Utilizing this method it was found that Φ_Δ_(**6**)=9.7 % whereas Φ_Δ_(**9**)=15.9 % (Figure 6). The enhancement of ^1^O_2_ generation exhibited in **9** can be rationalized as a result of increased conjugation throughout the molecule. To our knowledge, this is the first data regarding the generation of singlet oxygen for a TPE‐BODIPY conjugate of any kind.

Given that **9** has been shown to exhibit the AIE phenomenon, and the ability to generate ^1^O_2_; the question arises regarding the generation of ^1^O_2_ in the aggregated state. It is safe to propose that the Φ_Δ_ of the aggregated **9** would be significantly lower than that in the non‐aggregated state. Inherently, as a result of the processes involved, it's highly likely that the molecules closer to the center of these aggregates would not become excited, due to shielding from other molecules closer to the edge of the aggregate, and that those closer to the edge would likely quench via interaction with another molecule of close proximity – possibly further enhancing the AIE response. Thus, it would be only those molecules on the edge of the aggregate have the ability to generate ^1^O_2_ effectively, if at all.

The incorporation of multiple photosensitizers (PSs) into one system is not a novel idea in of itself,^[29]^ however as we have stated previously the decoration of a TPE with four PS moieties is. In this instance, logic would suggest that the greater the number of PS molecules, the greater the Φ_Δ_, assuming each PS acts as an individual PS. A key criterion in these systems is the retention of the properties of the singular PS, and subsequently that their affect is merely multiplied. The demonstration that these conjugates can generate ^1^O_2_ is a positive finding in terms of our eventual aim of using these conjugates as multi‐photosensitizer arrays. The differing values of Φ_Δ_ for the conjugates presented herein indicate that; for **6** the properties of the BODIPY are retained, whilst for compound **9** there is an additive affect as a result of the TPE‐BODIPY conjugation. Given these findings we propose that these molecules are suitable as multi‐photosensitizer arrays.

## Conclusions

In summary, we have presented the synthesis of novel tetra‐BODIPY appended TPE dyes in which the TPE and BODIPY moieties are linked either through a phenylene spacer (**6**) or phenyl‐acetylene spacer (**9**). Intermediates such as the tetra‐aldehyde **7** offer potential modular tectons for materials science applications. The structural differences between the linkages have been exemplified through the photophysical analyses undertaken; exhibiting a heightened AIE response upon the addition of an ethynyl spacer to the system, in the same mixed solvent system (THF/H_2_O), with emission bands at around 645 nm and 730 nm – yielding an aggregation induced dual emission, through differing intermolecular π−π stacking arrangements. Both of these systems were found to generation singlet oxygen, with Φ_Δ_(**6**)=9.7 % and Φ_Δ_(**9**)=15.9 %. These compounds (particularly **9**) present themselves as ideal candidates for applications such as intracellular imaging, with distinct intense emission bands, and also as photosensitizers given the high Φ_Δ_ values observed. This initial investigation indicates the possibility of further uses of the TPE core in the construction of spatially defined photosensitizer arrays for other applications i. e. light‐emitting systems and theranostics. Further investigations utilizing this concept of are underway with a variety of photosensitizers, and these findings will be published in due course.

## Experimental Section

**General Information**: Reactions involving moisture and/or air‐sensitive reagents were carried out in pre‐dried glassware and with standard Schlenk line techniques. All commercial reagents and anhydrous solvents were used as received from vendors (Fischer Scientific, and Sigma Aldrich). Dichloromethane (CH_2_Cl_2_) was dried with P_2_O_5_. Yields refer to chromatographically and spectroscopically (^1^H NMR) homogeneous material unless otherwise noted. Reactions were monitored by thin‐layer chromatography (TLC) and absorption spectroscopy. TLC was carried out on silica gel plates. Silica gel 60 *Merck, 230–400 mesh, or aluminum oxide (Brockmann Grade I) were used for flash column chromatography. Room temperature refers to 20–25 °C.

**Instrumentation**: Melting points are uncorrected and were measured with a Digital Stuart SPM 10 melting point apparatus. NMR spectra were recorded using Bruker DPX 400 and Agilent 400 were used to obtain ^1^H (400.13 MHz), ^13^C{^1^H} (100.61 MHz), ^19^F{^1^H} (376.60 MHz), and ^11^B (128.40 MHz) NMR spectra, and a Bruker AV 600 was employed for ^1^H (600.13 MHz) and ^13^C{^1^H} (150.90 MHz) NMR spectra. NMR spectroscopy was carried out at room temperature. Chemical shifts are given in ppm and referenced to the residual peak of the deuterated NMR solvent. The assignment of the signals was confirmed by 2D spectra (COSY, HMBC, HSQC). MALDI TOF spectra were acquired using a Waters Maldi Q‐Tof Premier. The instrument was operated in positive or negative mode as required. The laser operated at 337 nm. Samples were run using DCTB (*trans*‐2‐[3‐(4‐tert‐butylphenyl)‐2‐methyl‐2‐propenylidene]malononitrile) as a MALDI matrix. The instrument was calibrated using PEG. The internal lock mass used was [Glu] Fibrinopetide B. MassLynx 4.1 software was used to carry out the analysis. ESI mass spectra were acquired in positive or negative modes as required, using a Micromass time‐of‐flight mass spectrometer (TOF), or a Bruker mircoOTOF−Q II spectrometer interfaced to a Dionex UltiMate 3000 LC. APCI experiments were carried out on a Bruker microOTOF‐Q III spectrometer interfaced to a Dionex Ultimate 3000 C or direct insertion probe in positive or negative modes. UV/Vis spectra were recorded in solutions using a Specord 250 spectrophotometer from Analytik Jena (1 cm path length quartz cell).

**Singlet oxygen studies**: The photo‐irradiation of the samples was performed in quartz cuvettes (2×1×1 cm) under irradiation via a polychromatic light source (Philips, 15 V–150 W lamp), equipped with a 400 nm cut‐off filter (Schott GG 400) and a 532 nm diode‐pumped solid state green laser system (CW532‐04, average intensity of 10 mW cm^−2^). The temperature of the sample was maintained at 18 °C using a Peltier element (Cary Peltier 1×1 cell holder). Relative singlet oxygen (^1^O_2_) yields (*Φ*_TPE‐BODIPY_) were calculated from the degradation slopes of the 1,3‐diphenylisobenzofuran (DPBF) conversion in the presence of different photosensitizers. The absorbance of DPBF molecule was adjusted to 1.0 at 417 nm in an air‐saturated solvent mixture then the corresponding photosensitizer was added to the solution. The solutions were irradiated from 0.5–1 h, and absorption spectra were recorded at *t*=0 s and intervals of 100 s. A subsequent decrease in the absorbance of DPBF was observed after each irradiation. Singlet oxygen experiments were repeated twice and *Φ*_TPE‐_ values were observed in a standard deviation range of ± 0.05.

Compounds **2 a**, **2 b**, **3**, and **4** were prepared according to literature procedures.^[21]^

**1,1,2,2‐Tetrakis(4′‐(di(1*H*‐pyrrol‐2‐yl)methyl)‐[1,1′‐biphenyl]‐4‐yl)ethylene (5)**. A solution of **4** (100 mg, 0.133 mmol, 1.0 equiv.) in pyrrole (3 mL) was degassed with argon for 5 min. The solution was stirred for 15 min at room temperature under argon in the presence of InCl_3_ (118 mg, 0.534 mmol, 5.0 equiv.). The reaction mixture was diluted with CH_2_Cl_2_ (50 mL) and washed with 0.1 M NaOH solution, water and brine (1×25 mL each, in that order). The solvent was removed under reduced pressure to give a dark green oil crude product which was purified via flash column chromatography (SiO_2_, EtOAc:Hex, 1 : 1, *v*/*v*). The title compound was obtained as a grey solid upon rotary evaporation (90 mg, 0.074 mmol, 56 %). M.p.=>205–207 °C (dec.); R_*f*_=0.51 (SiO_2_, EtOAc:Hex, 1 : 1, *v*/*v*);^1^H NMR (400 MHz, CDCl_3_): *δ*=7.87 (s, 4H), 7.48 (d, *J*=7.6 Hz, 8H), 7.34 (d, *J*=8.0 Hz, 8H), 7.20 (t, *J*=8.1 Hz, 8H), 7.17–7.11 (m, 8H), 6.66 (s, 8H), 6.15 (d, *J*=2.7 Hz, 8H), 5.91 (s, 8H), 5.44 (s, 4H) ppm; ^13^C NMR (100 MHz, CDCl_3_): *δ*=142.8, 141.1, 140.5, 139.2, 138.5, 132.3, 132.0, 128.8, 127.0, 126.2, 117.3, 108.4, 107.2, 43.6 ppm; HRMS (MALDI) calcd. for C_86_H_68_N_8_ [M^+^]: 1212.5567; found 1212.5504.

**1,1,2,2‐Tetrakis(4′‐(4,4‐difluoro‐4‐bora‐3 a,4 a‐diaza‐*s*–indacene)‐[1,1′‐biphenyl]‐4‐yl)ethylene (6)**. A solution of **5** (50 mg, 0.04 mmol) in CH_2_Cl_2_ (5 mL) was degassed with Ar for 5 min. DDQ (37 mg, 0.164 mmol) was added and the reaction mixture was stirred for 5 min. Et_3_N (80 μL, 0.618 mmol) was added and the solution was stirred for a further 3 min, before addition of BF_3_ ⋅ OEt_2_ (86 μL, 0.64 mmol). The reaction mixture was stirred for 35 min at room temperature and monitored *via* TLC. The reaction was quenched with water and the organic phase extracted with CH_2_Cl_2_ (3×50 mL) The organic phase was washed twice with water (25 mL), dried (MgSO_4_) and solvent evaporated to yield a crude green product. This was purified *via* flash column chromatography (EtOAc:Hex, 1 : 2, *v*/*v*) to yield an orange solid (19 mg, 32 %). M.p.=208–210 °C (dec.); R_*f*_=0.68 (SiO_2_, EtOAc:^*n*^Hex, 1 : 1, *v*/*v*); ^1^H NMR (400 MHz, CDCl_3_): *δ*=7.94 (s, 8H), 7.75 (d, *J*=8.2 Hz, 8H), 7.65–7.60 (m, 8H), 7.53 (d, *J*=8.2 Hz, 8H), 7.29 (d, *J*=8.2 Hz, 8H), 6.96 (d, *J*=4.0 Hz, 8H), 6.54 (d, *J*=2.9 Hz, 8H) ppm; ^13^C NMR (101 MHz, CDCl_3_): *δ*=143.1(7), 143.1(4), 142.5(1), 142.5(0), 132.1, 132.0, 128.6, 121.9, 117.8, 117.6, 108.7, 108.3, 107.5, 44.0 ppm; ^11^B NMR (128.4 MHz, CDCl_3_): *δ*=0.30 (t, ^1^ *J*_B‐F_=28.7 Hz, 4B) ppm; ^19^F NMR (376.5 MHz, CDCl_3_): *δ*=−145.06 ppm (q, ^1^J_F‐B_=28.6 Hz, 8F) ppm; HRMS (MALDI) calcd. for C_86_H_56_B_4_F_8_N_8_ [M^+^]: 1396.4872; found 1396.4917.

**1,1,2,2‐Tetrakis(4‐(4′‐formylphenylethynyl)phenyl)ethylene (7)**. To an oven and flame dried Schlenk tube was added; **2 b** (215 mg, 501.7 μmol), 4‐iodobenzaldehyde (1.0 g, 4.310 mmol, 8.6 eq.), PPh_3_ (86 mg, 0.327 mmol), Pd(PPh_3_)_4_ (65 mg, 56.3 μmol), and CuI (53 mg, 278.3 μmol). The solids were dried under high vacuum (<0.1 mbar) for 2 h. Added to this was anhydrous 1,4‐dioxane (8 mL) and anhydrous Et_3_N (2 mL). The mixture underwent three freeze‐pump‐thaw cycles before being heated at 100 °C for 25 h. Upon cooling to RT the mixture was passed through a pad of silica (EtOAc) and excess solvent was removed at reduced pressure. The product was adsorbed onto silica (THF) and purified via column chromatography (silica, EtOAc/Hex, 1/2, *v/v*). Excess solvent was removed under reduced pressure, and the residue was sonicated with Et_2_O to yield the product as a brick orange solid (170 mg, 201.2 μmol, 40 %). M.p.=158–160 °C (dec.); R_*f=*_0.24 (SiO_2_, EtOAc : Hex 1 : 2, *v/v*); ^1^H NMR (CDCl_3_, 400 MHz): *δ*=10.01 (s, 4H), 7.86 (d, *J*=8.3 Hz, 8H), 7.64 (d, *J*=8.2 Hz, 8H), 7.35 (d, *J*=8.3 Hz, 8H), 7.06 (d, *J*=8.3 Hz, 8H) ppm; ^13^C NMR (CDCl_3_, 101 MHz): *δ*=191.5, 143.6, 141.3, 135.6, 132.2, 131.7, 129.8, 129.6, 121.4, 93.4, 85.9 ppm; HRMS (APCI) calcd. for C_62_H_36_O_4_ [M]^−^: 844.2630; found: 844.2619.

**1,1,2,2‐Tetrakis(4‐((4‐(di(1*H*‐pyrrol‐2‐yl)methyl)phenyl)ethynyl)phenyl)ethylene (8)**. To a round bottom flask was added **7** (102 mg, 120.7 μmol) and freshly distilled pyrrole (5 mL). The solution was purged with argon and InCl_3_ was added (134 mg, 605.8 μmol) and the mixture was stirred under argon until TLC indicated complete consumption of **7** (c.a. 20 mins). The reaction mixture was diluted with CH_2_Cl_2_ (50 mL) and subsequently washed with H_2_O, 0.1 M NaOH and brine (1×25 mL each, in that order). The organic extract was dried (MgSO_4_) and excess solvent was removed at reduced pressure. The product was purified via column chromatography (SiO_2_ EtOAc/Hex, 1/1, *v/v*) to yield the desired product as a grey solid (40 mg, 30.5 μmol, 25 %). M.p.=>300 °C (dec.) ; R_*f*_=0.65 (EtOAc:Hex 1 : 1, *v/v*); ^1^H NMR (CDCl_3_, 400 MHz): *δ*=7.93 (br s, 8H), 7.45 (d, *J*=8.2 Hz, 8H) 7.30 (d, *J*=8.3 Hz, 8H), 7.18 (d, *J*=8.2 Hz, 8H), 7.01 (d, *J*=8.3 Hz, 8H), 6.70–6.71 (m, 8H), 6.15–6.18 (m, 8H), 5.91 (s, 8H), 5.47 (s, 4H) ppm; ^13^C NMR (CDCl_3_, 400 MHz): *δ*=143.2, 143.1, 142.5(1), 142.5(0), 132.1, 132.0, 128.6, 121.9, 117.8, 117.6, 108.7, 108.3, 107.5, 44.0 ppm; HRMS (MALDI‐TOF): calcd. for C_94_H_68_N_8_ [M^+^]: 1308.5567; found 1308.5552.

**1,1,2,2‐Tetrakis(4‐((4‐(4,4‐difluoro‐4‐bora‐3 a,4 a‐diaza‐*s*–indacene)phenyl)ethynyl)phenyl)ethene (9)**. A solution of **8** (50 mg, 0.04 mmol) in CH_2_Cl_2_ (5 mL) was degassed with argon for 5 min. DDQ (32 mg, 0.164 mmol) was added and the reaction mixture was stirred for 5 min. Et_3_N (75 μL, 0.62 mmol) was added and the solution was stirred for a further 3 min before addition of BF_3_ ⋅ OEt_2_ (78 μL, 0.64 mmol). The reaction mixture was stirred for 35 min at room temperature and monitored via TLC. The reaction was quenched with H_2_O and the organic phase extracted with CH_2_Cl_2_ (2×25 mL). The organic phase was washed with water (2×25 mL), dried (MgSO_4_), and the solvent evaporated to yield a crude green product which was purified via flash column chromatography (EtOAc:Hex, 1 : 2, *v*/*v*) to yield an orange solid (11 mg, 7.37 μmol, 19 %). M.p.=>150 °C (dec.); R_*f*_=0.72 (SiO_2_, EtOAc:Hex, 1 : 1, *v*/*v*);^1^H NMR (400 MHz, CDCl_3_): *δ*=7.96 (s, 8H), 7.68 (d, *J*=8.3 Hz, 8H), 7.57 (d, *J*=8.3 Hz, 8H), 7.52 (d, *J*=8.2 Hz, 8H), 7.22 (d, *J*=8.2 Hz, 8H), 6.95 (d, *J*=4.1 Hz, 8H), 6.56 (m, 8H) ppm; ^11^B NMR (128.4 MHz, CDCl_3_): *δ*=0.28 (t, ^1^ *J*_B‐F_=28.7 Hz, 4B) ppm.; ^19^F NMR (376.5 MHz, CDCl_3_): *δ*=−145.08 ppm (q, ^1^ *J*_F‐B_=28.6 Hz, 8F) ppm; ^13^C NMR (101 MHz, CDCl_3_): *δ*=144.5, 141.6, 134.9, 133.6, 132.2, 131.8(9), 131.8(6), 131.7, 131.5, 130.8, 130.7, 129.3, 126.4, 118.9 ppm; HRMS (MALDI) calcd. for C_102_H_74_B_3_F_6_N_8_NaO_4_ [M‐BF_2_+2H+2EtOAc+Na^+^]: 1644.5914; found 1644.5953.

**Crystal structure determinations**. Crystals were grown via slow evaporation at room temperature from saturated solutions of MeOH (**3**) and CH_2_Cl_2_/F_3_CCO_2_H (**4**)

Single crystal X‐ray diffraction data for all compounds were collected on a Bruker APEX Kappa Duo diffractometer by using Incoatec IμS Cu*K*_α_ (*λ*=1.54178 Å) radiation. Crystals were mounted on a MiTeGen MicroMount and collected at 100(2) K by using an Oxford Cryosystems Cobra low temperature device. Data were collected by using omega and phi scans and were corrected for Lorentz and polarization effects by using the APEX software suite.^[30]^ Using Olex2, the structures were solved with the XT structure solution program, using the intrinsic phasing solution method and refined against |F_2_| with XL using least‐squares minimization.^[30]^ Hydrogen atoms were generally placed in geometrically calculated positions and refined using a riding model. All images were rendered using Olex2.

Crystal Data for **3**: C_23_H_26_OSi_2_ (M=374.62 g mol^−1^): orthorhombic, space group P*ccn* (no. 56), *a*=33.9086(9) Å, *b*=5.5681(2) Å, *c*=11.6573(3) Å, α=β=γ=90°, V=2200.97(11) Å^3^, Z=4, T=100(2) K, μ(Cu K_α_)=1.514 mm^−1^, *D*_calc_=1.131 g cm^−3^, 15541 reflections measured (2.606°≤2θ≤69.836°), 2057 unique (*R*_int_=0.0423, *R*_sigma_=0.0251) which were used in all calculations. The final *R*_1_ was 0.0404 (I >2σ(I)) and *wR*_2_ was 0.1152 (all data).

Crystal data for **4**: C_54_H_36_O_4_ (M=748.83 g mol^−1^): monoclinic, space group C*2/c* (no. 15), *a*=35.8085(13) Å, *b*=9.0237(3) Å, *c*=34.1519(11) Å, α=γ=90°, β=120.7872(19) °, V=9480.2(6) Å^3^, Z=8, T=100(2) K, μ(Cu K_α_)=0.514 mm^−1^, *D*_calc_=1.049 g cm^−3^, 35951 reflections measured (2.873°≤2θ≤58.986°), 6752 unique (*R*_int_=0.0602, *R*_sigma_=0.0486) which were used in all calculations. The final *R*_1_ was 0.1168 (I>2σ(I)) and *wR*_2_ was 0.3682 (all data). Sample showed poor diffraction, resolution was limited to d=0.9 Angstroms. Two terminal carboxy phenyl groups were modelled as disordered in two locations using rigid groups, occupancies C37, 56 %; C37B 44 % and C51, 85 %; C51b, 15 %. Refined with restraints (DFIX, SIMU, RIGU and ISOR). It was not possible to refine the solvents in the lattice voids and their contribution to the diffraction data was removed using the SQUEEZE routine in PLATON.^[32]^ The solvent accessible volume (SAV) is 2025 Å^3^ and there are 636 electrons found in this SAV. This is a mixture of CH_2_Cl_2_ and F_3_CCO_2_H.

## Supporting Information

Spectroscopic data of all compounds, singlet oxygen production measurements, and X‐ray crystallographic data.

Deposition Numbers 2063490 (for **3**) and 2063491 (for **4**) contain the supplementary crystallographic data for this paper. These data are provided free of charge by the joint Cambridge Crystallographic Data Centre and Fachinformationszentrum Karlsruhe Access Structures service www.ccdc.cam.ac.uk/structures.

## Conflict of interest

The authors declare no conflict of interest.

## Supporting information

As a service to our authors and readers, this journal provides supporting information supplied by the authors. Such materials are peer reviewed and may be re‐organized for online delivery, but are not copy‐edited or typeset. Technical support issues arising from supporting information (other than missing files) should be addressed to the authors.

Supporting Information
