# Supplementary material for: Synthesis and Properties of BODIPY Appended Tetraphenylethylene Scaffolds as Photoactive Arrays
Source: European J Org Chem. 2021 Aug 4;2021(29):4136–43. doi: 10.1002/ejoc.202100629 (PMC8457078; doi:10.1002/ejoc.202100629)
Supplement: Supplementary file 1 — Supporting Information [file EJOC-2021-4136-s001.pdf]

European Journal of Organic Chemistry

Supporting Information

Synthesis and Properties of BODIPY Appended Tetraphenylethylene Scaffolds as Photoactive Arrays

Harry C. Sample, Ganapathi Emandi, Brendan Twamley, Nitika Grover, Bhavya Khurana, Vincent Sol, and Mathias O. Senge*

SUPPORTING INFORMATION

Contents

1. NMR Data for Compound 5 2

2. NMR Data for Compound 6 4

3. NMR Data for Compound 7 8

4. NMR Data for Compound 8 10

5. NMR Data for Compound 9 12

6. Mass Spectrometry Data 16

7. AIE Emission Studies for Compound 6 21

8. AIE Emission Studies for Compound 9 23

9. Singlet Oxygen Studies 25

10. Crystallography 30

1. NMR Data for Compound 5

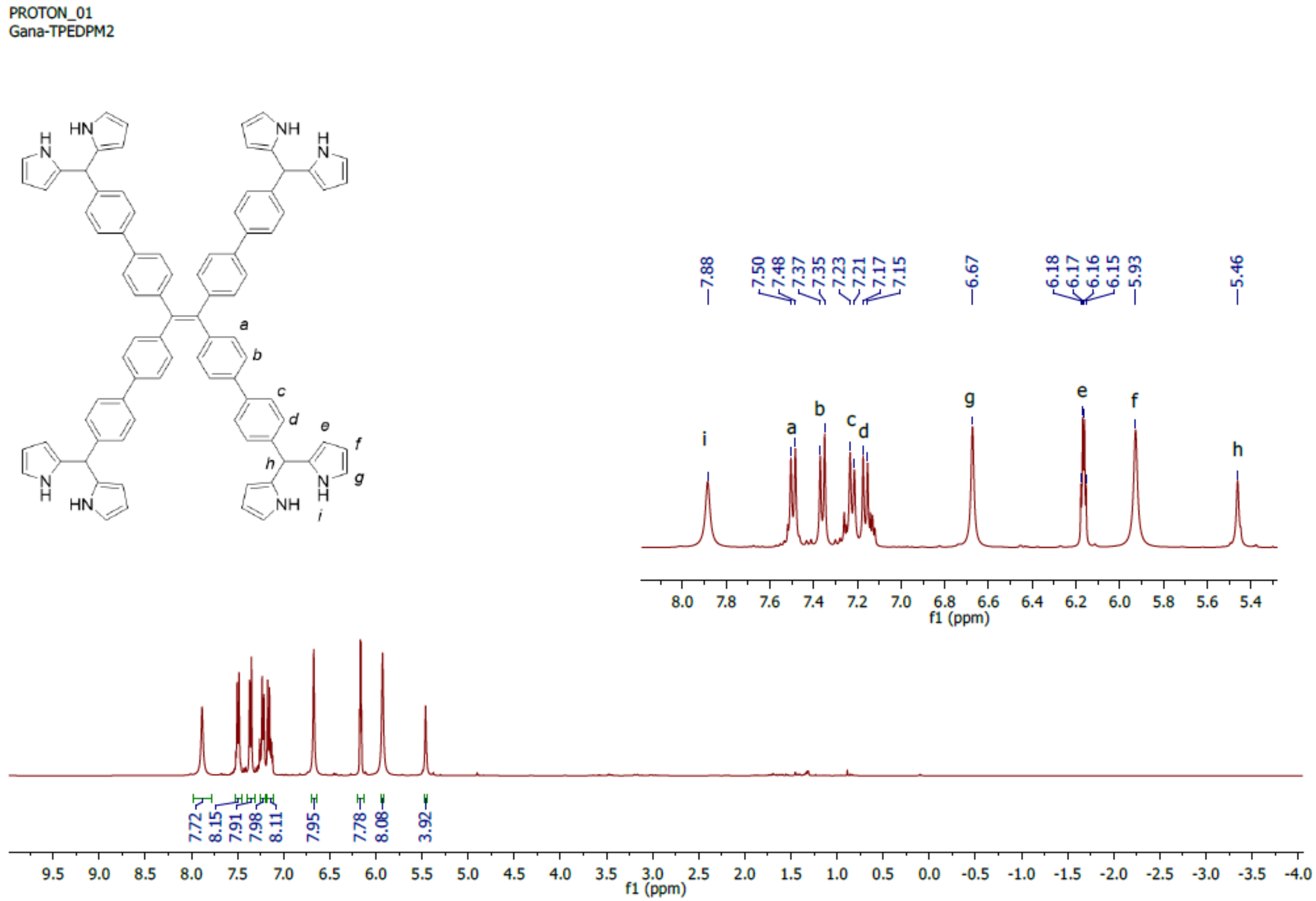

Figure S1: ^{1}H NMR spectrum of **5** in $CDCl_3$ at 25 ºC (400 MHz)

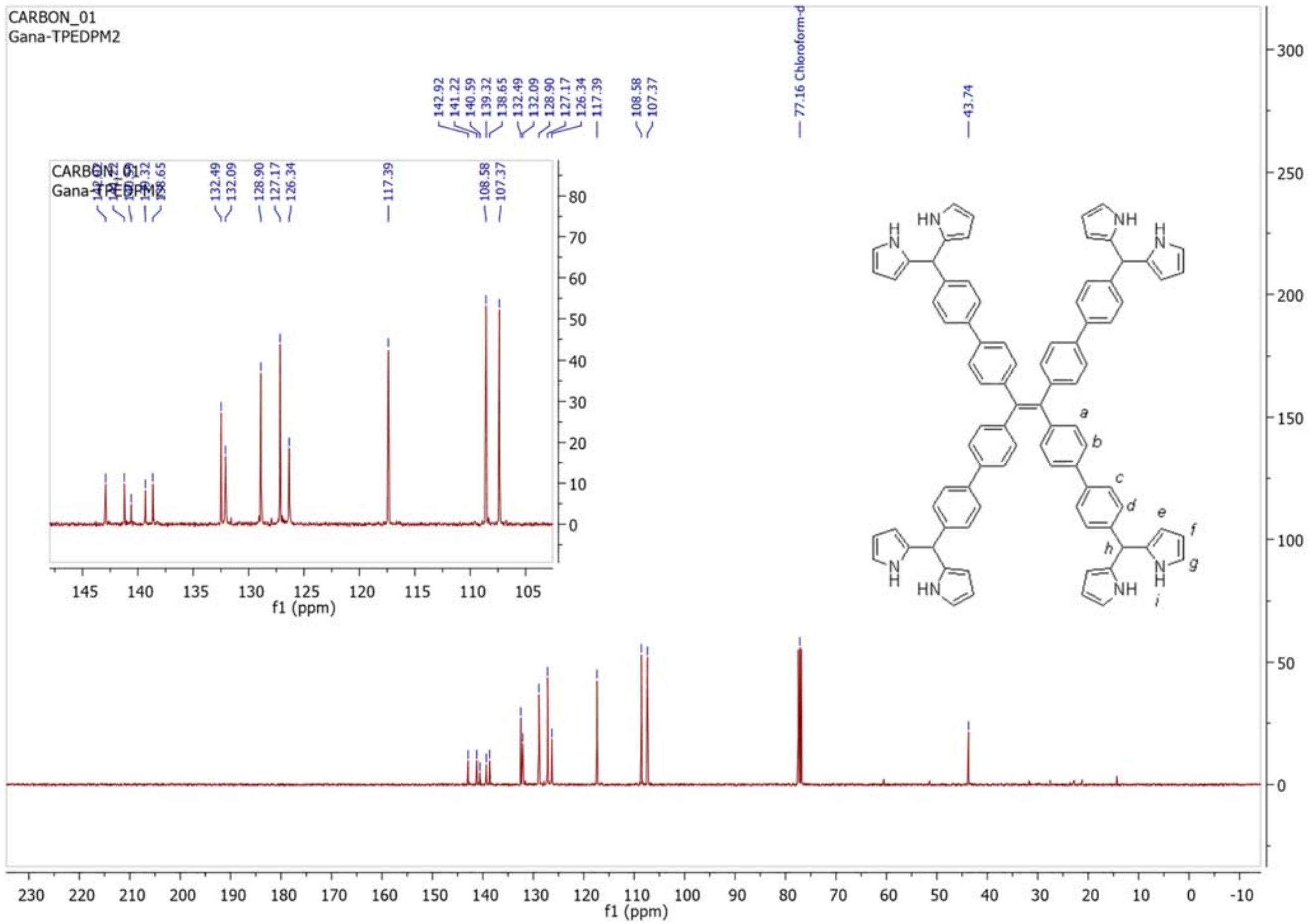

Figure S2: ^{13}C NMR spectrum of **5** in $CDCl_3$ at 25 ºC (101 MHz)

2. NMR Data for Compound 6

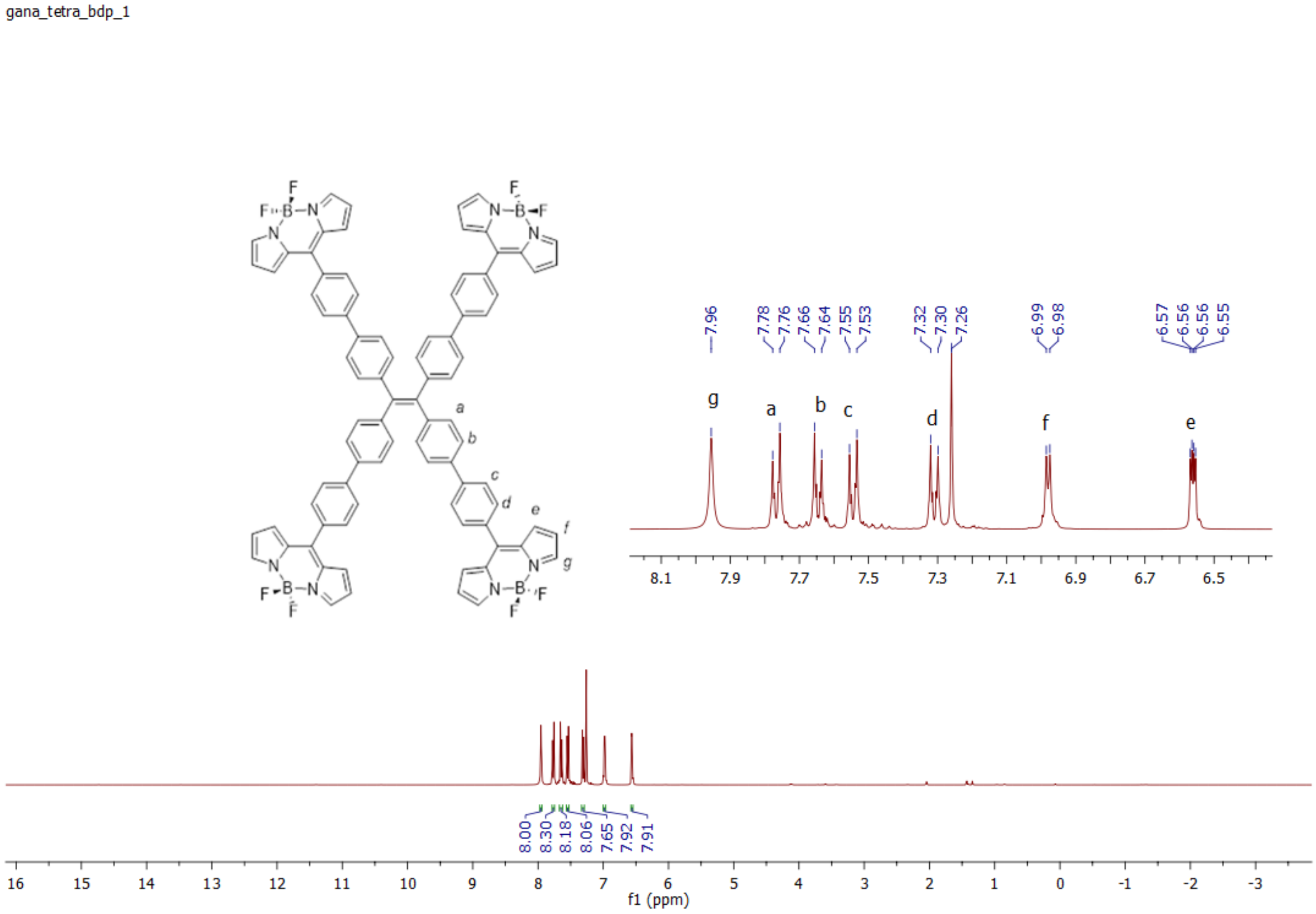

Figure S3: ^{1}H NMR spectrum of **6** in $CDCl_3$ at 25 ºC (400 MHz).

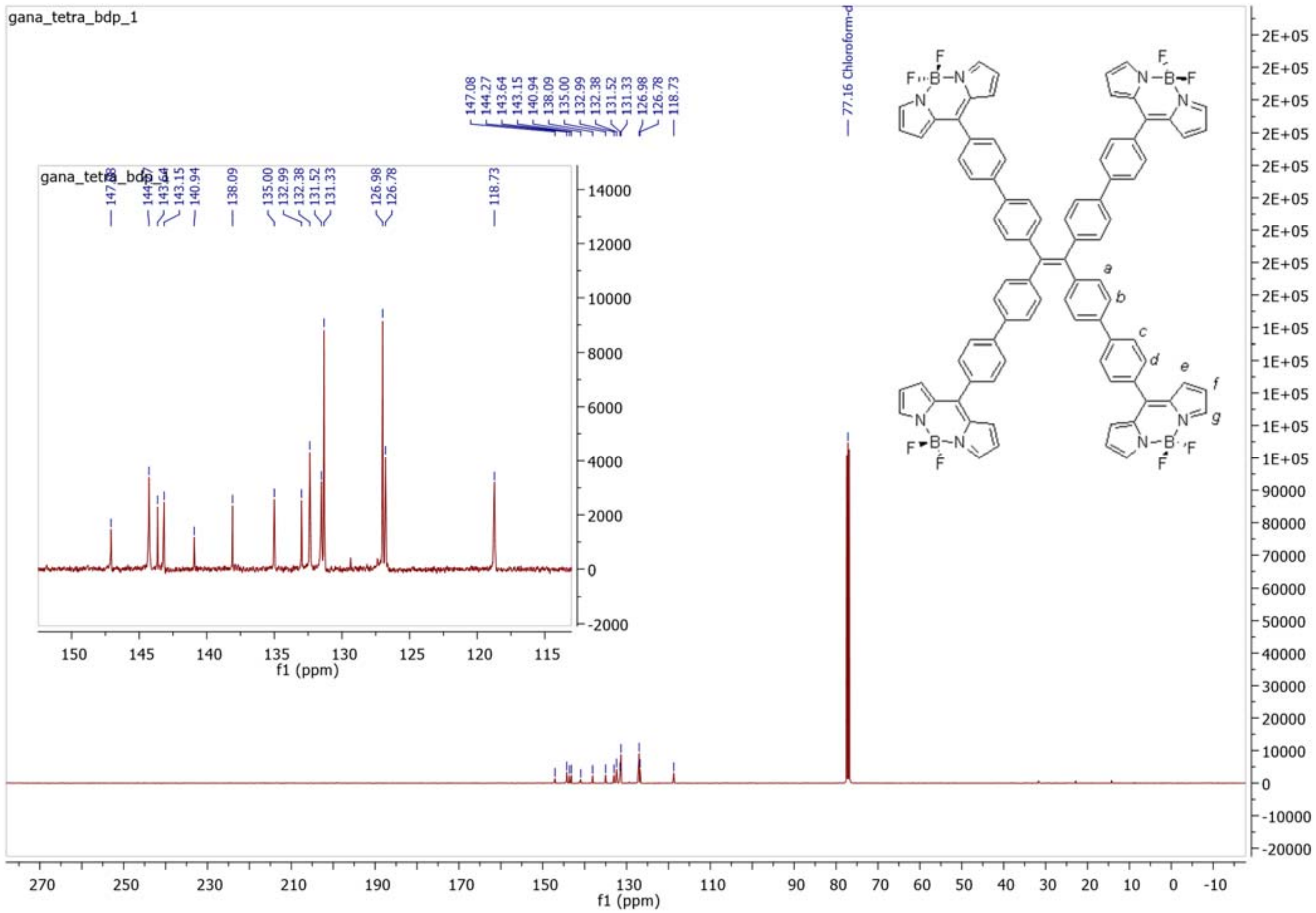

Figure S4: ^{13}C NMR spectrum of **6** in $CDCl_3$ at 25 ºC (101 MHz)

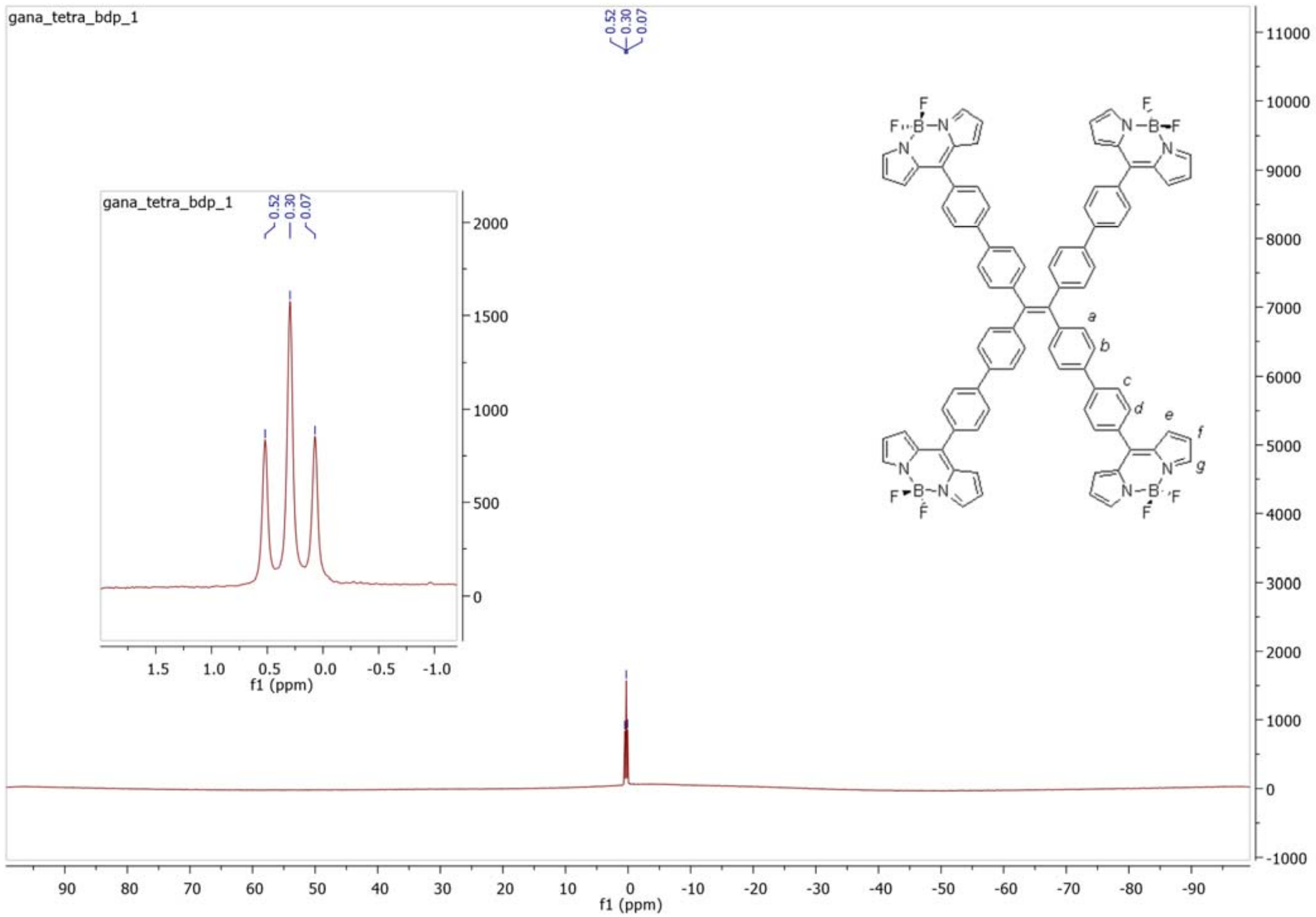

Figure S5: ^{11}B NMR spectrum of **6** in $CDCl_3$ at 25 ºC (128 MHz).

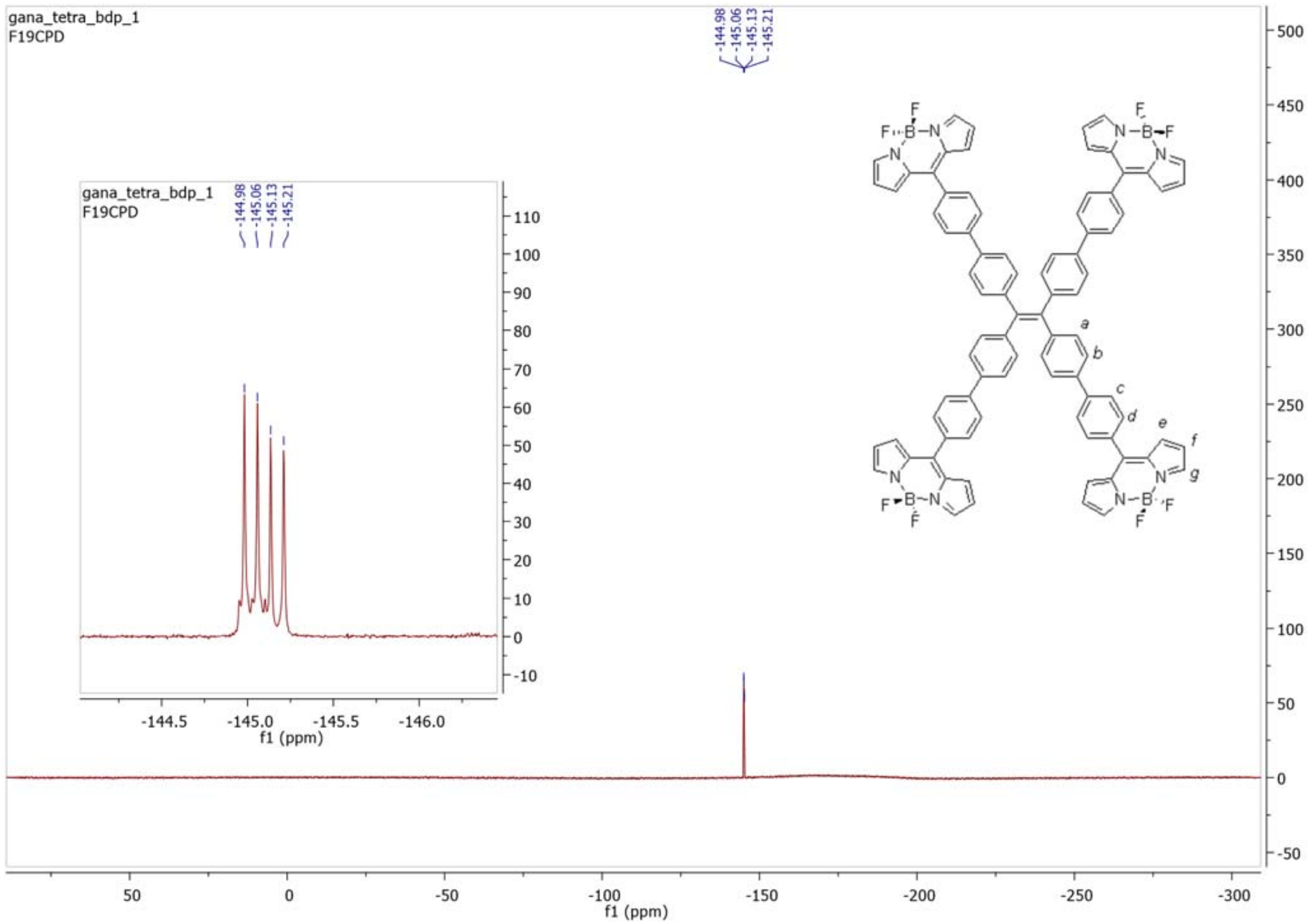

Figure S6: ^{19}F NMR spectrum of **6** in $CDCl_3$ at 25 ºC (377 MHz).

3. NMR Data for Compound 7

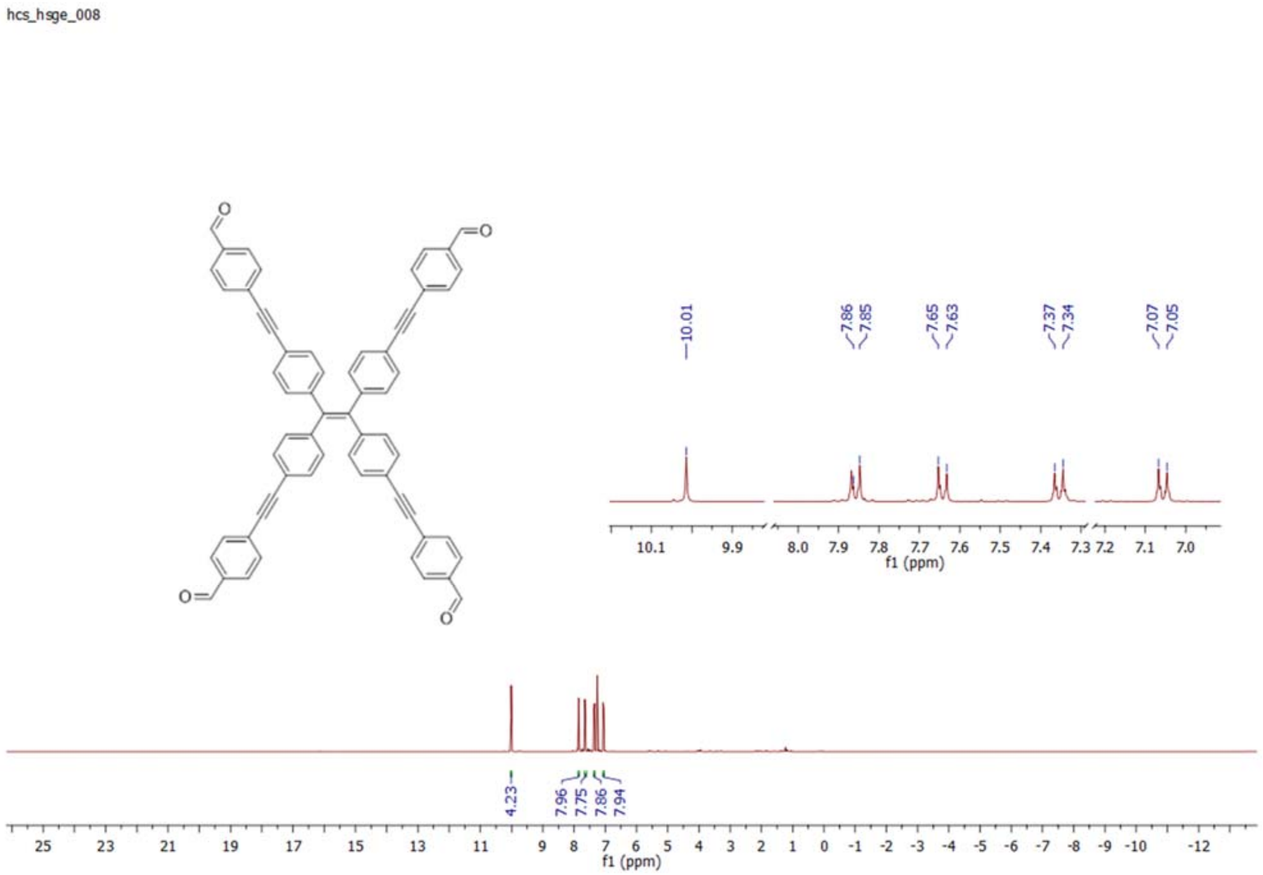

Figure S7: ^{1}H NMR spectrum of **7** in $CDCl_3$ at 25 ºC (400 MHz)

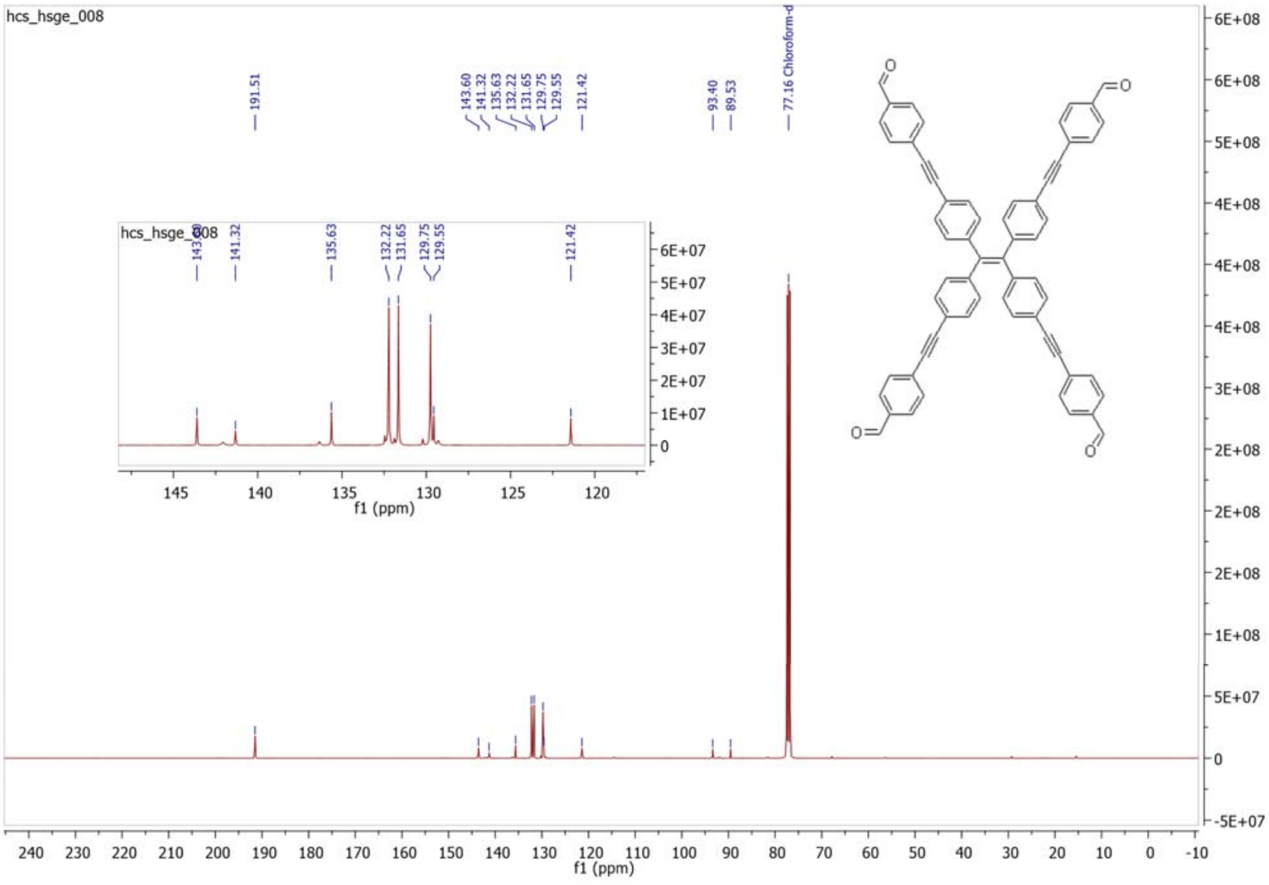

Figure S10: ^{13}C NMR spectrum of **7** in $CDCl_3$ at 25 ºC (101 MHz).

4. NMR Data for Compound 8

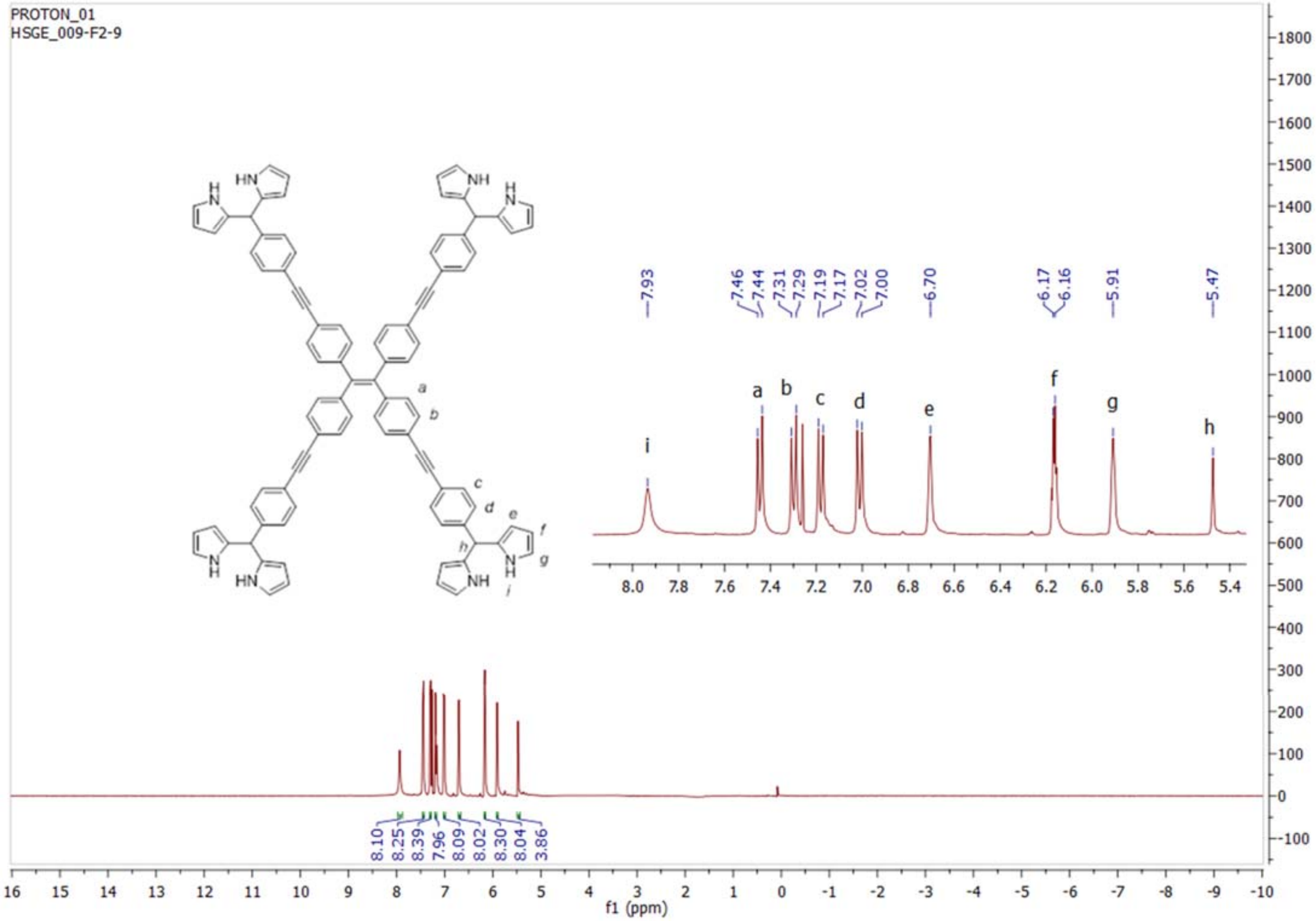

Figure S11: ^1H NMR spectrum of **8** in $CDCl_3$ at 25 ºC (400 MHz).

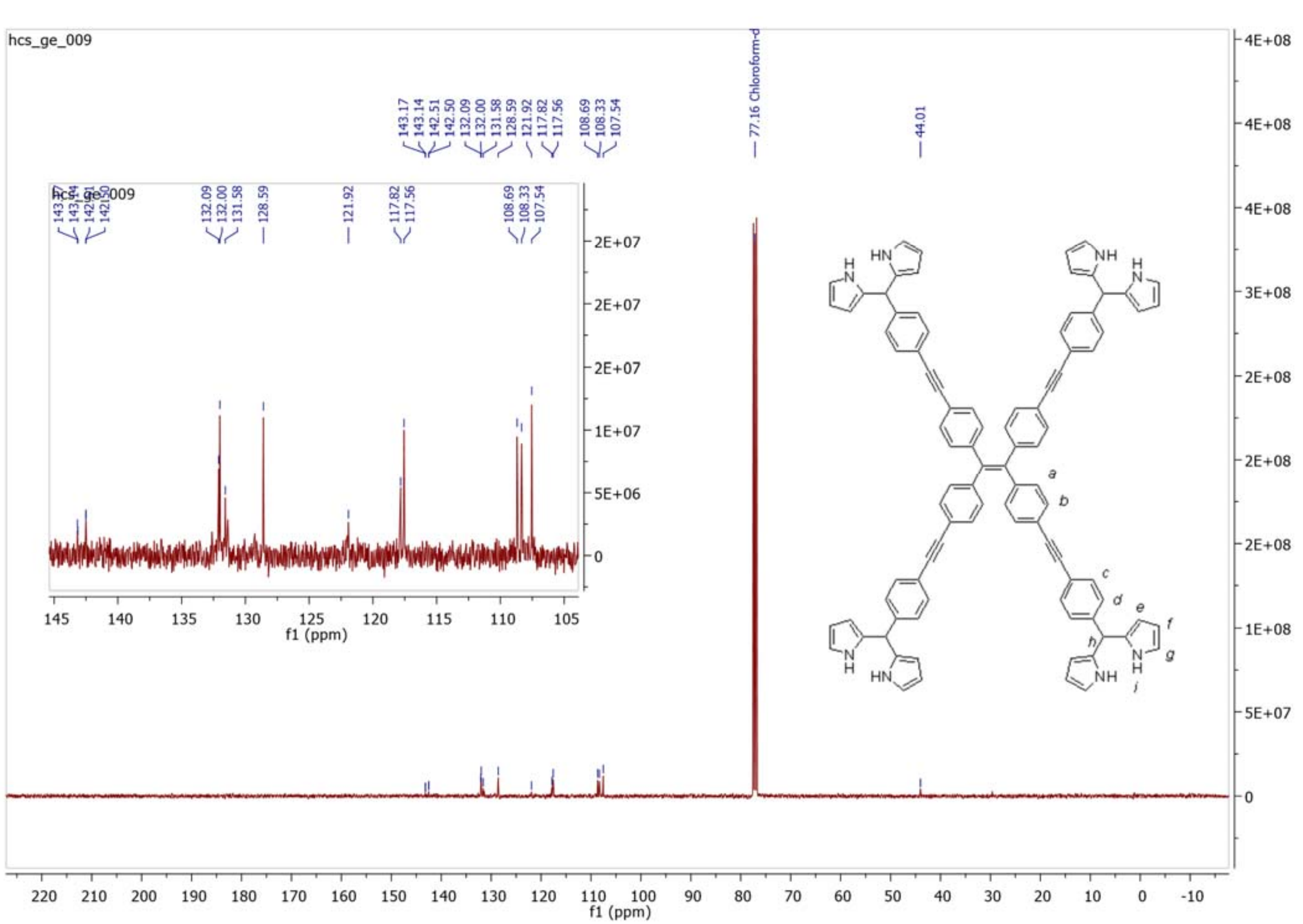

Figure S12: ^{13}C NMR spectrum of **8** in $CDCl_3$ at 25 ºC (101 MHz).

5. NMR Data for Compound 9

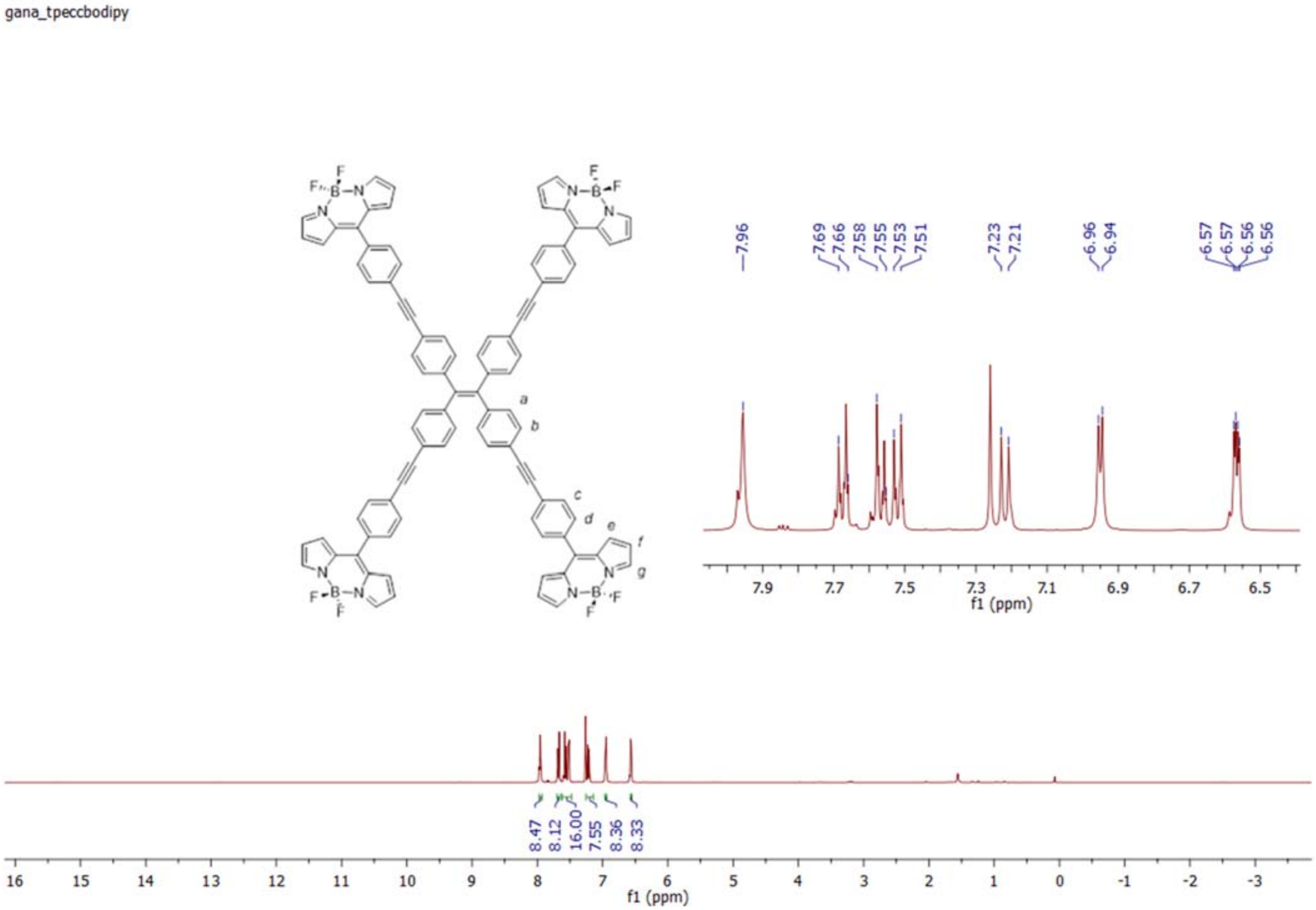

Figure S13: ^{1}H NMR spectrum of **9** in $CDCl_3$ at 25 ºC (400 MHz).

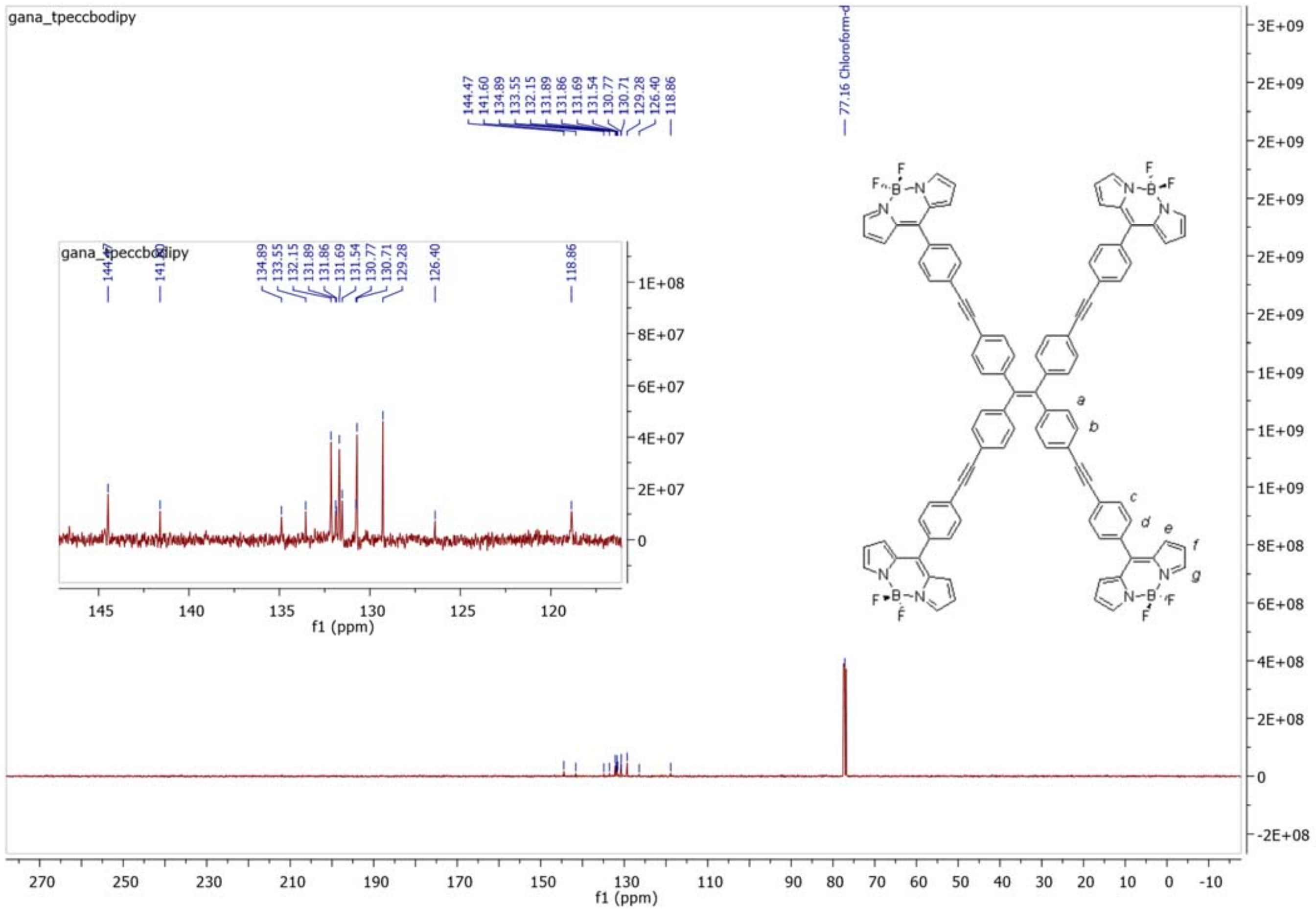

Figure S14: ^{13}C NMR spectrum of **9** in $CDCl_3$ at 25 ºC (101 MHz).

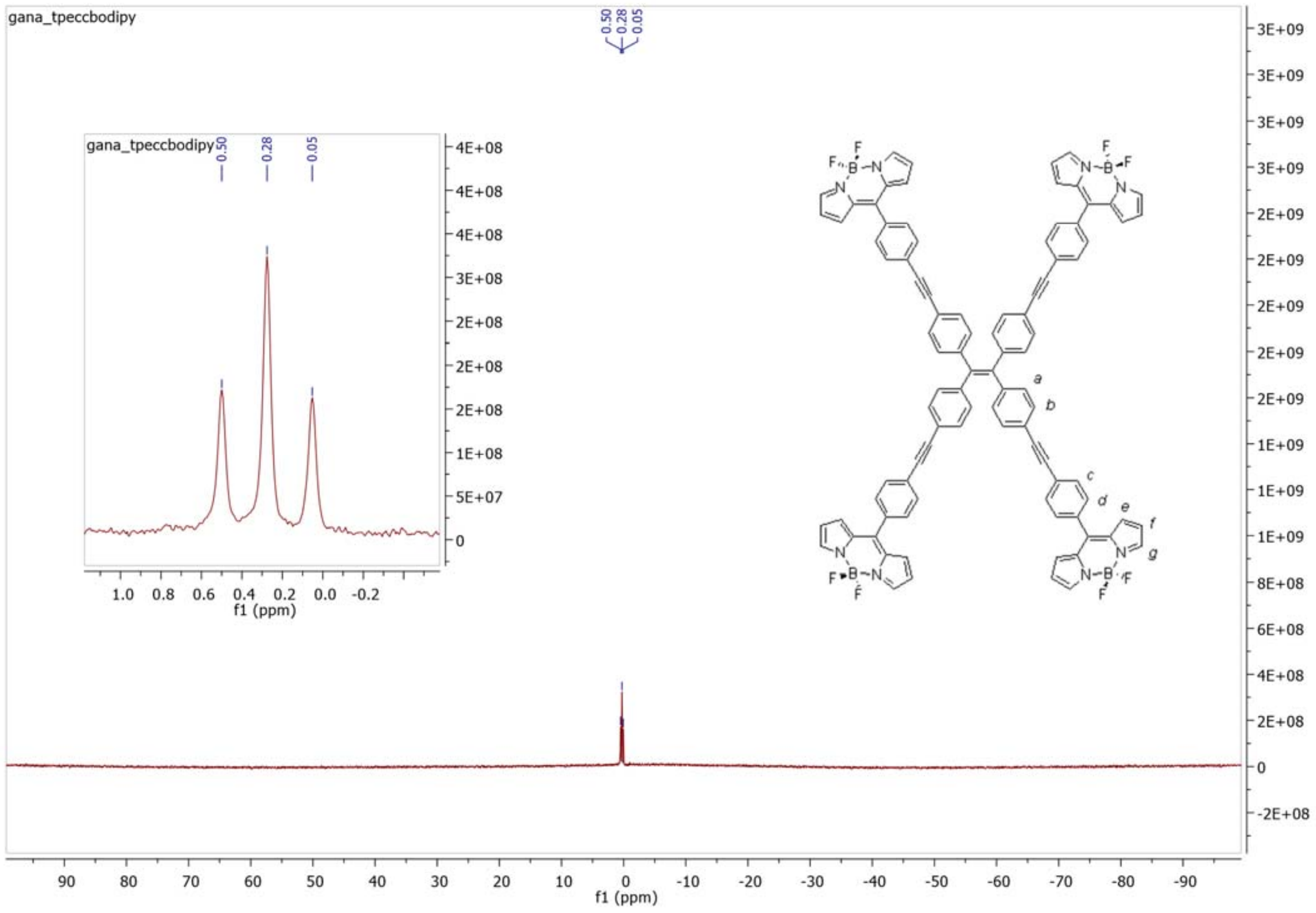

Figure S15: ^{11}B NMR spectrum of **9** in $CDCl_3$ at 25 ºC. (128 MHz)

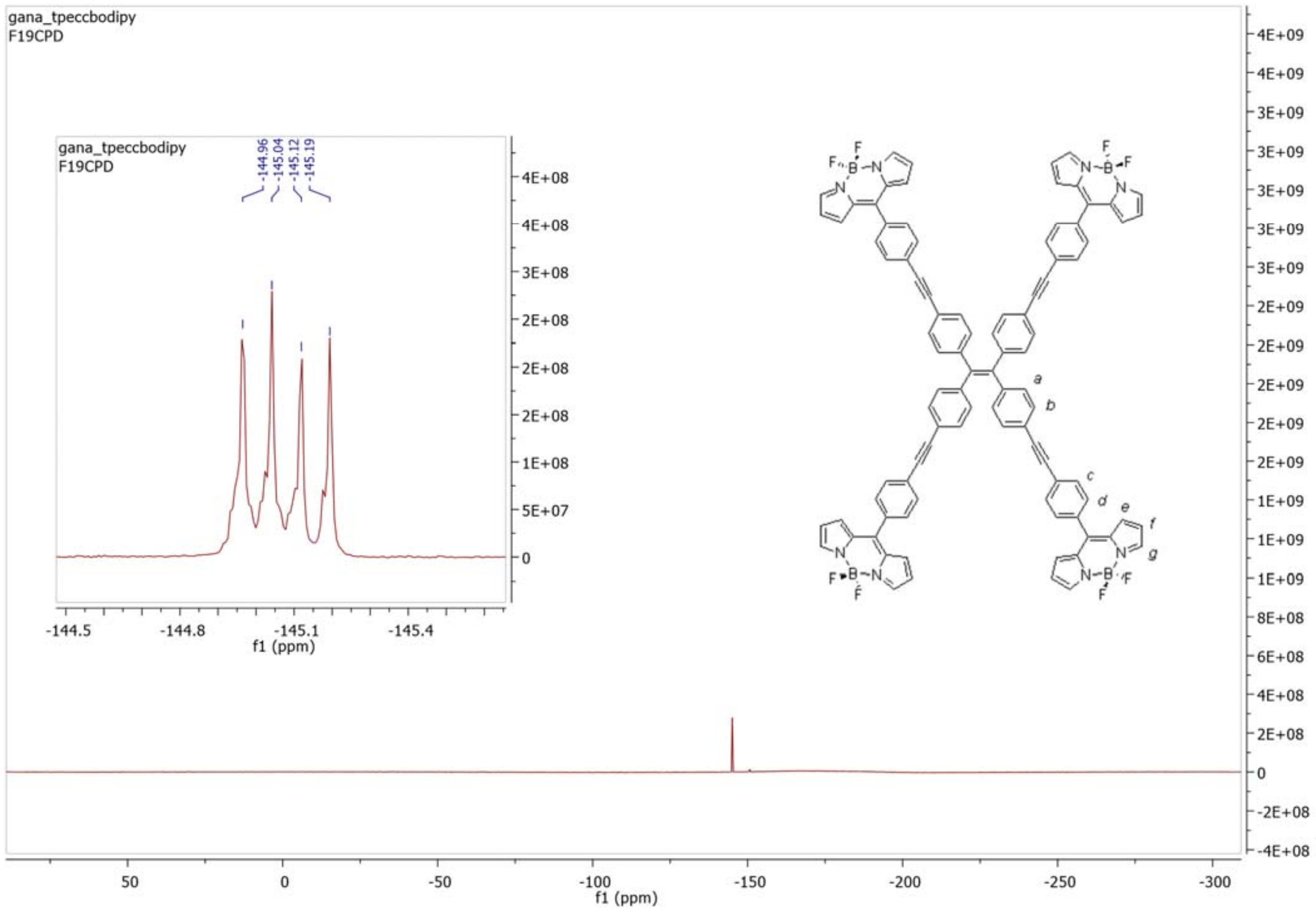

Figure S16: ^{19}F NMR spectrum of **9** in $CDCl_3$ at 25 ºC (377 MHz).

6. Mass Spectrometry Data

Elemental Composition Report

Page 1

Single Mass Analysis

Tolerance = 100.0 PPM / DBE: min = -1.5, max = 400.0
Element prediction: Off
Number of isotope peaks used for i-FIT = 5

Monoisotopic Mass, Odd and Even Electron Ions
1 formula(e) evaluated with 1 results within limits (up to 10 closest results for each mass)
Elements Used:
C: 0-86 H: 0-68 N: 0-8

Ganapathi (MSe), Gana-tetraDPM
Q-TOF20190307MF02 41 (0.756) AM (Cen,8, 80.00, Ht,10000.0,1570.68,0.70); Sm (SG, 2x3.00); Sb (15,10.00); Cm (7:94-38:43) TOF MS LD+
2.39e+003

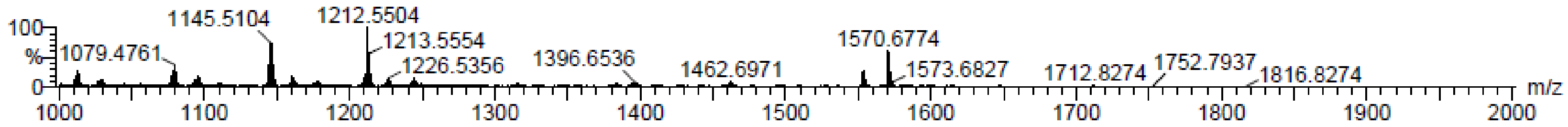

Minimum:				-1.5			
Maximum:		5.0	100.0	400.0			
Mass	Calc. Mass	mDa	PPM	DBE	i-FIT	i-FIT (Norm)	Formula
1212.5504	1212.5567	-6.3	-5.2	57.0	96.3	0.0	C86 H68 N8

Figure S17: MALDI-TOF (DCTB Matrix) Mass Spectrum of **5**.

Elemental Composition Report

Single Mass Analysis

Tolerance = 100.0 PPM / DBE: min = -1.5, max = 400.0
Element prediction: Off
Number of isotope peaks used for i-FIT = 5

Monoisotopic Mass, Odd and Even Electron Ions
257 formula(e) evaluated with 1 results within limits (up to 10 closest results for each mass)
Elements Used:
C: 0-86 H: 0-56 N: 0-8 F: 0-8 B: 0-4

Ganapathi (MSe), Gana-tetraBDP-1
Q-TOF20190218MF002 283 (5.243) AM (Cen,8, 80.00, Ht,10000.0,1570.68,0.70); Sm (SG, 2x3.00); Sb (15,10.00); Cm (274:339) TOF MS LD+ 1.85e+003

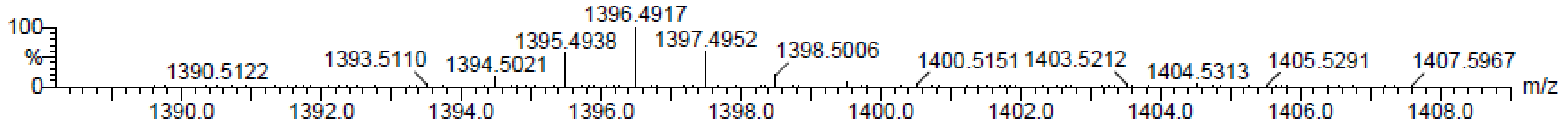

Mass	Calc. Mass	mDa	PPM	DBE	i-FIT	i-FIT (Norm)	Formula
Minimum:				-1.5			
Maximum:		5.0	100.0	400.0			
1396.4917	1396.4872	4.5	3.2	61.0	120.6	0.0	C86 H56 N8 F8 B4

Figure S18: MALDI-TOF (DCTB Matrix) Mass Spectrum of **6**.

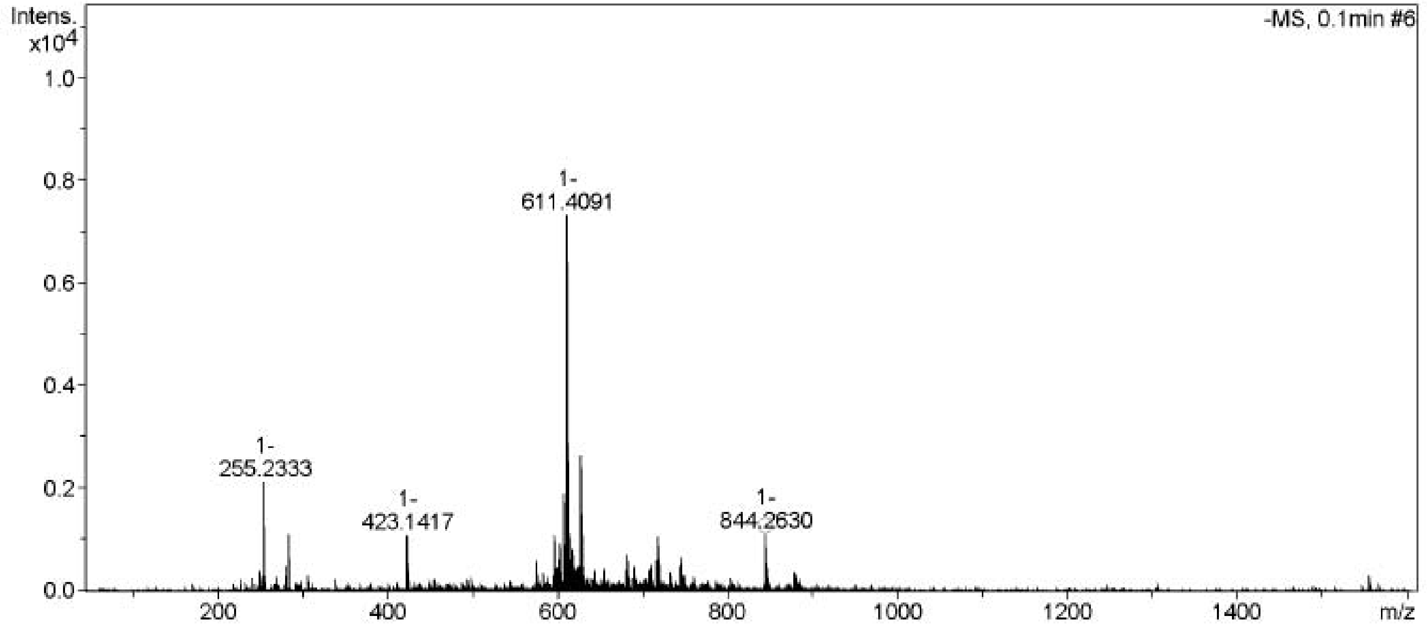

Meas. m/z	#	Ion Formula	m/z	err [mDa]	err [ppm]	rdb	N-Rule	e⁻ Conf	mSigma
844.263034	1	$C_{62}H_{36}O_4$	844.261908	-1.1	-1.3	45.0	ok	odd	147.1

Figure S19: APCI Mass Spectrum of **7** (THF).

Elemental Composition Report

Page 1

Single Mass Analysis

Tolerance = 50.0 PPM / DBE: min = -1.5, max = 400.0
Element prediction: Off
Number of isotope peaks used for i-FIT = 5

Monoisotopic Mass, Odd and Even Electron Ions
1 formula(e) evaluated with 1 results within limits (up to 10 closest results for each mass)
Elements Used:
C: 0-94 H: 0-68 N: 0-8
Harry Sample (MSe), HSGE_009
Q-TOF20210319GH003 12 (0.222) AM (Cen,8, 80.00, Ht,10000.0,1570.68,0.70); Sm (SG, 2x3.00); Sb (15,10.00); Cm (6:79)

TOF MS LD+
1.95e+003

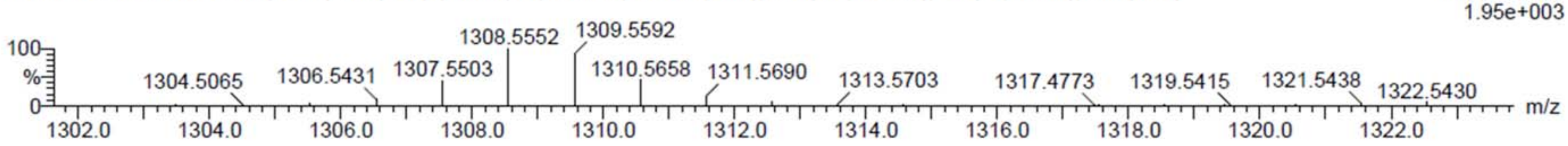

Minimum:				-1.5			
Maximum:		5.0	50.0	400.0			

Mass	Calc. Mass	mDa	PPM	DBE	i-FIT	i-FIT (Norm)	Formula
1308.5552	1308.5567	-1.5	-1.1	65.0	80.3	0.0	C94 H68 N8

Figure S20: MALDI-TOF (DCTB Matrix) Mass Spectrum of **8**

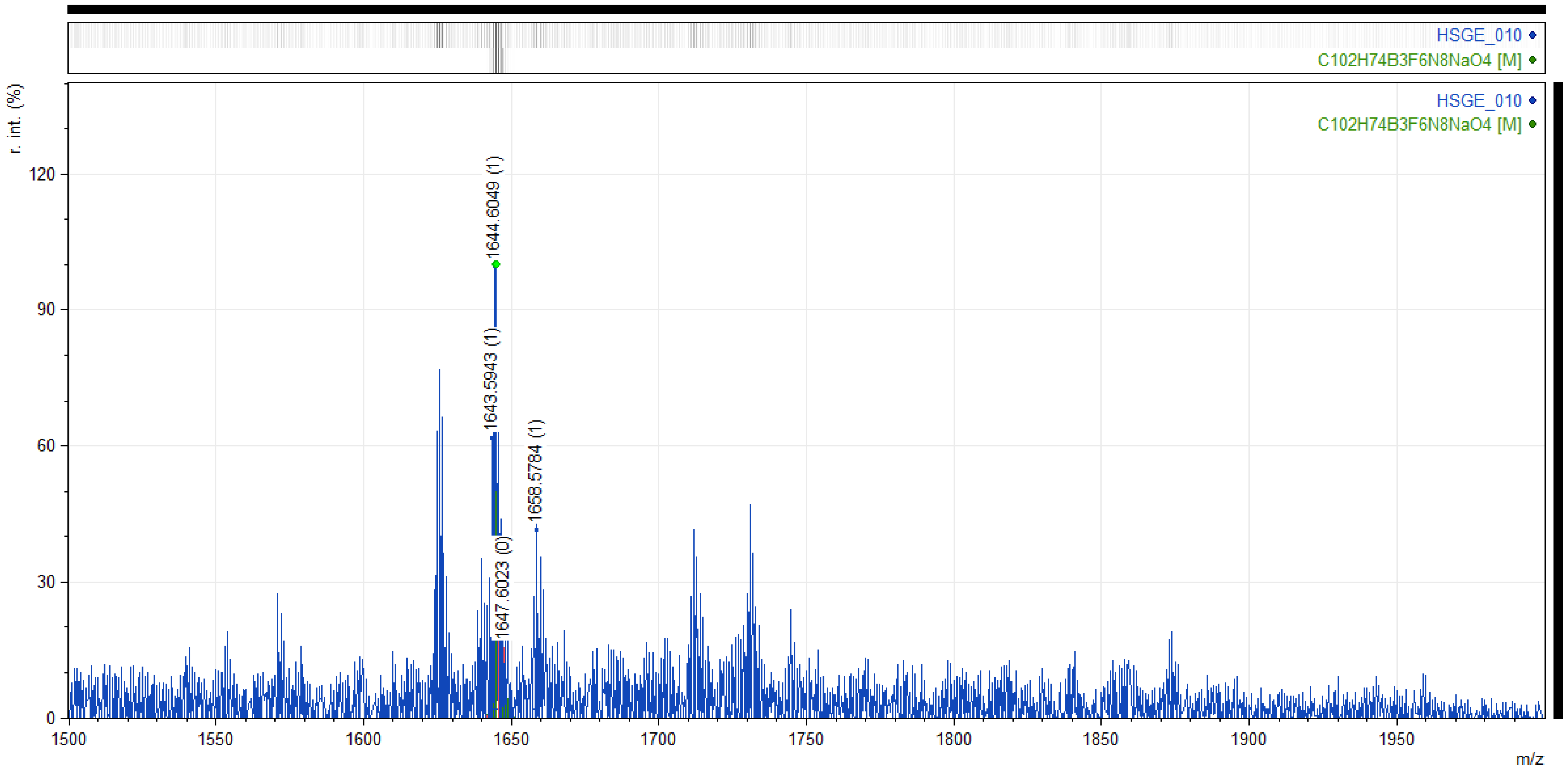

Figure S21. MALDI-TOF (DCTB Matrix) Mass Spectrum of **9**.

7. AIE Emission Studies for Compound 6

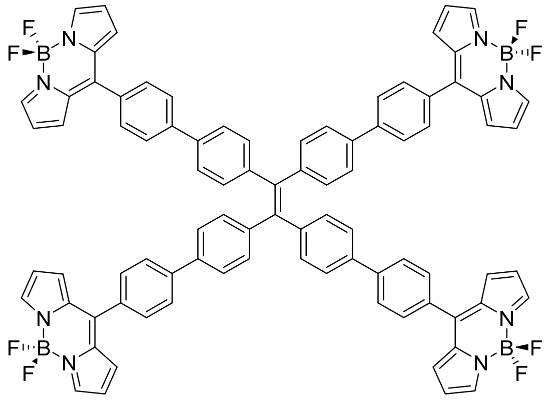

6

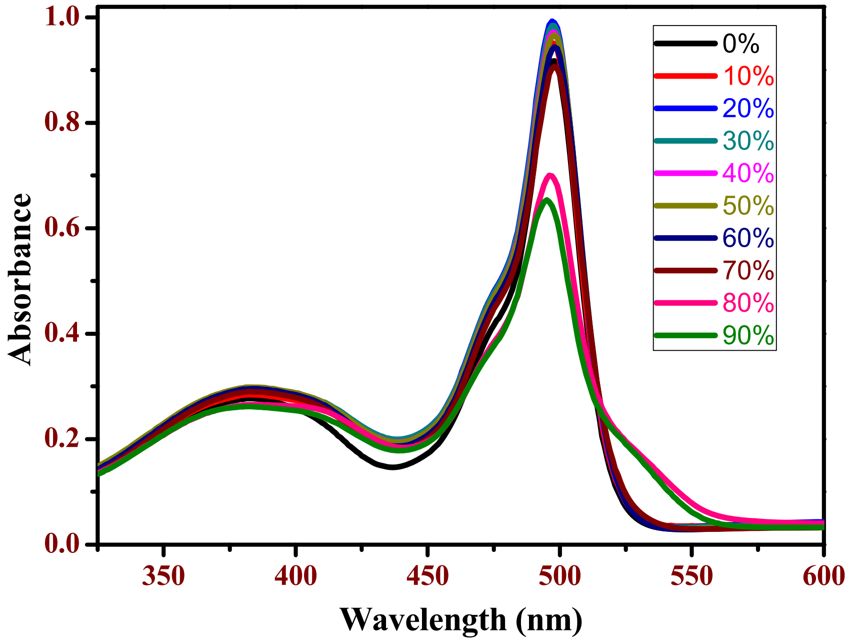

a)

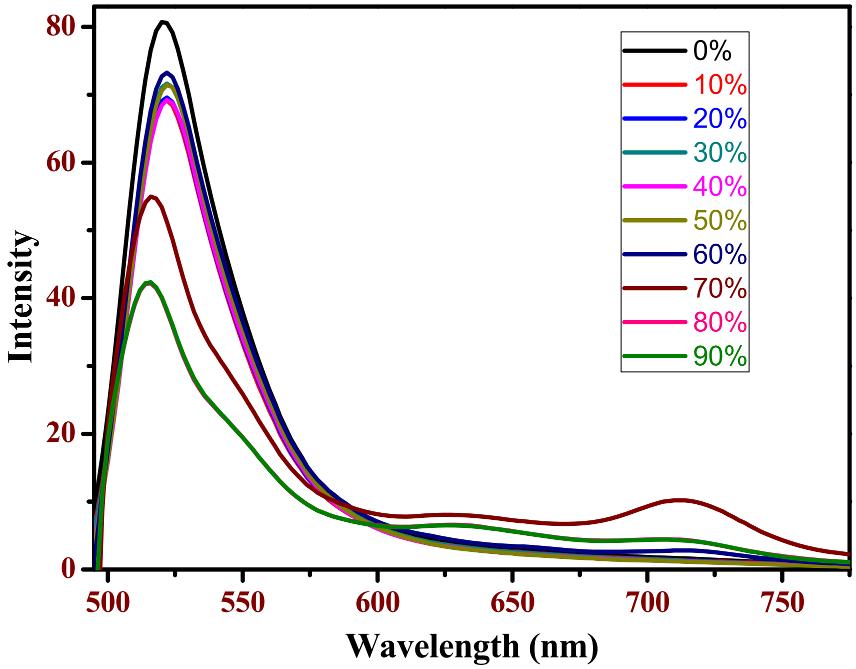

b)

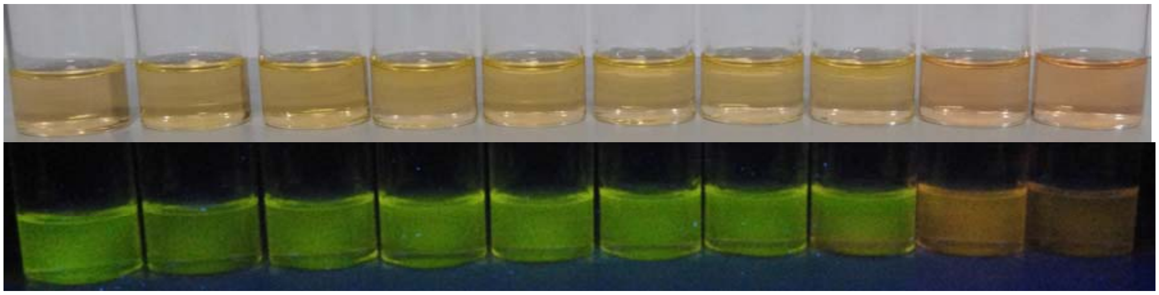

c)

Figure S22: Top: Structure of TPE-BODIPY conjugate **6**; a) overlayed absorption spectra of **6** in mixtures of THF/H_2O with varying %H_2O; b) overlayed emission spectra of **6** in mixtures of THF/H_2O with varying %H_2O; c) Photograph of the solutions of **6** in the varying THF/H_2O solutions under visible light (top) and excitation at 360 nm (bottom).

8. AIE Emission Studies for Compound 9

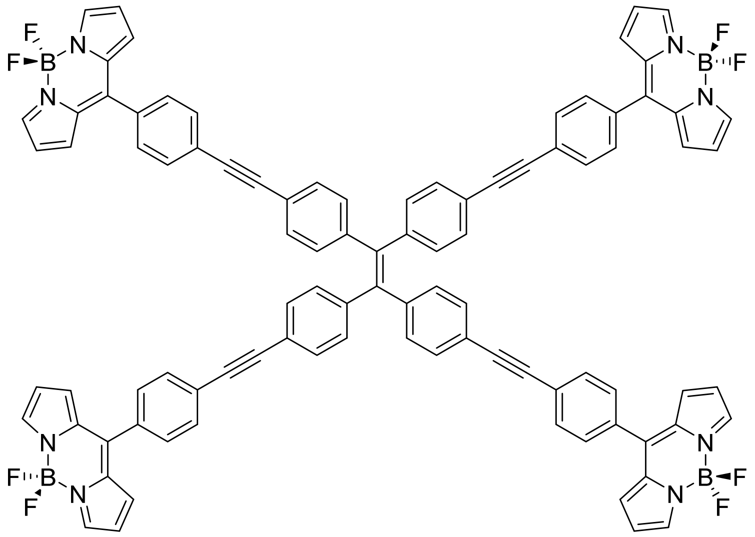

9

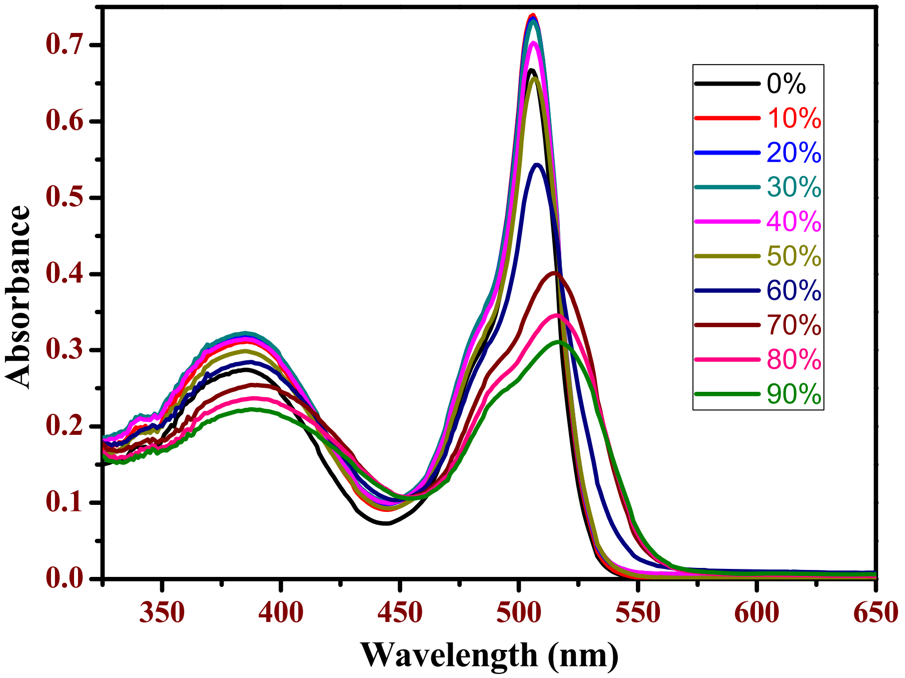

a)

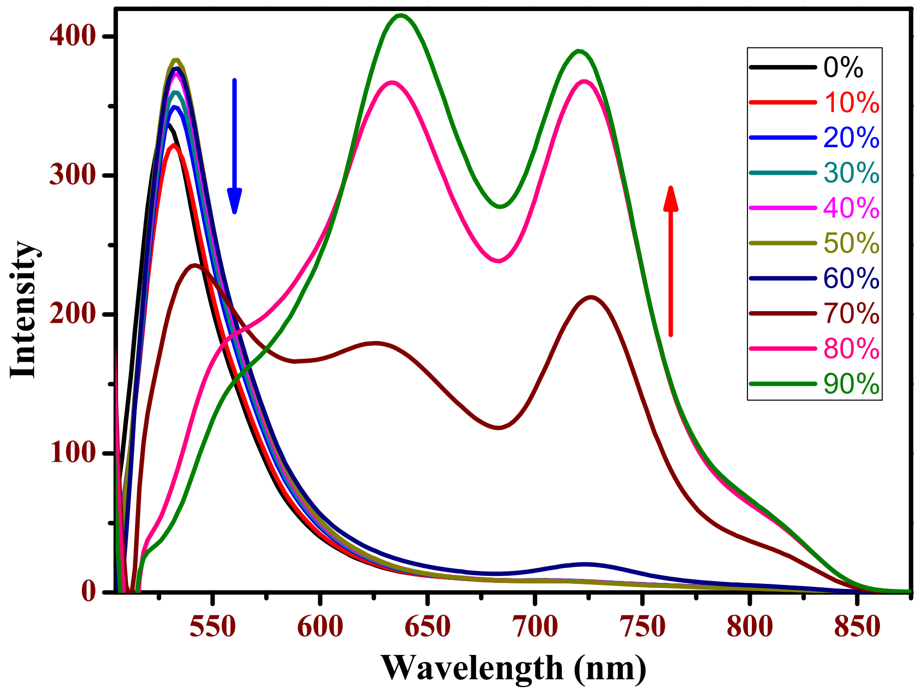

b)

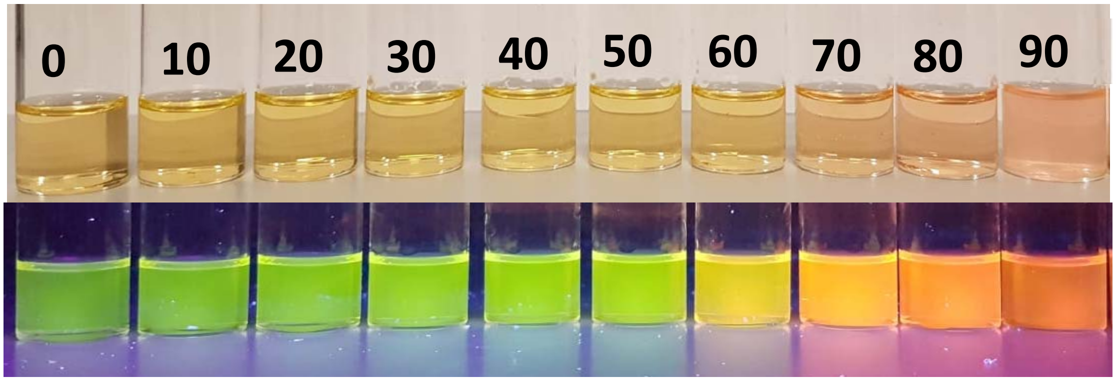

c)

Figure S23: Top: Structure of TPE-BODIPY conjugate **9**; a) overlayed absorption spectra of 9 in mixtures of THF/H_2O with varying %H_2O; b) overlayed emission spectra of **9** in mixtures of THF/H_2O with varying %H_2O; c) Photograph of the solutions of **9** in the varying THF/H_2O solutions under visible light (top) and excitation at 360 nm (bottom).

9. **Singlet oxygen studies**

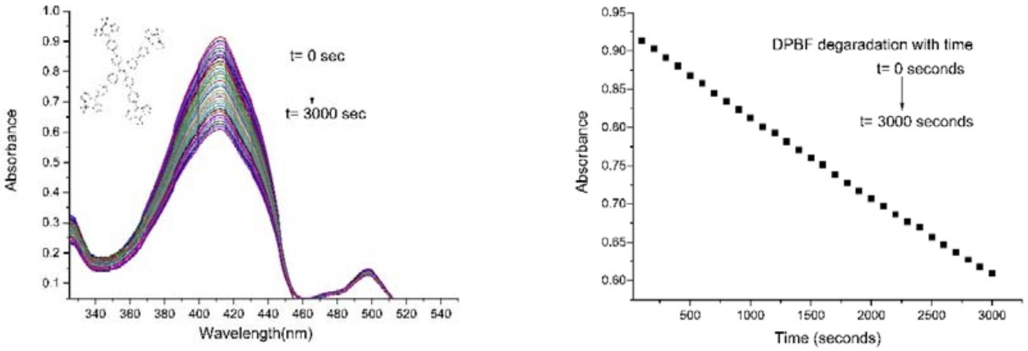

Figure S24: Left: UV-Vis spectra of DPBF degradation in the presence of **6** in DCM:MeOH (1:1) as a function of laser irradiation time (polychromatic light source). Right: DPBF consumption measured at 417 nm over time for H_2TPP in DCM:MeOH (1:1).

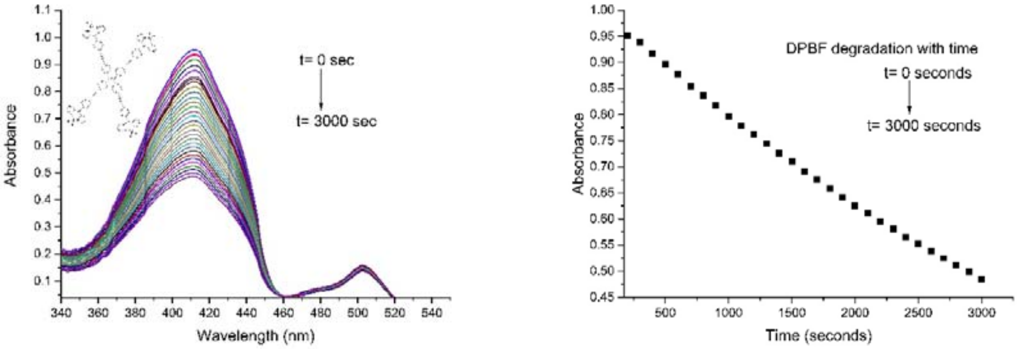

Figure S25: Left: UV-Vis spectra of DPBF degradation in the presence of **9** in DCM:MeOH (1:1) as a function of laser irradiation time (polychromatic light source). Right: DPBF consumption measured at 417 nm over time for H_2TPP in DCM:MeOH (1:1).

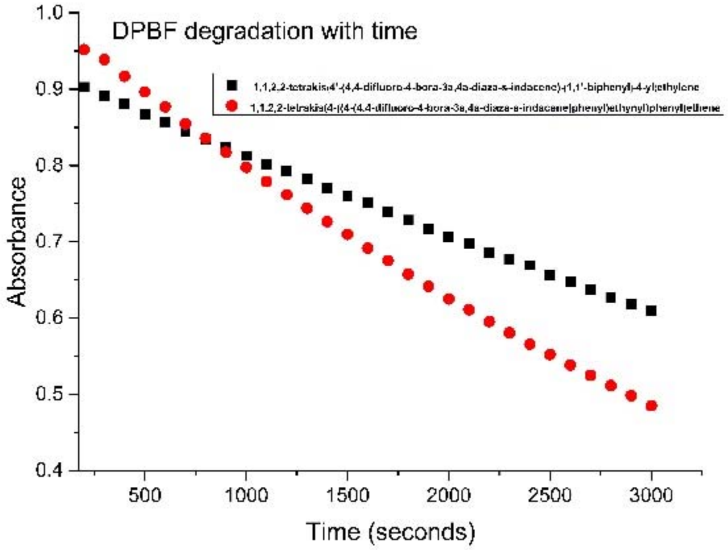

Figure S26: Overlay of the DPBF degradation curves for **6** (black) and **9** (red).

10. Crystallography

Single crystal X-ray diffraction data for all compounds were collected on a Bruker APEX Kappa Duo diffractometer by using Incoatec IμS CuKα (λ = 1.54178 Å) radiation. Crystals were mounted on a MiTeGen MicroMount and collected at 100(2) K by using an Oxford Cryosystems Cobra low temperature device. Data were collected by using omega and phi scans and were corrected for Lorentz and polarization effects by using the APEX software suite [1]. Using Olex2, [2] the structures were solved with the XT [3] structure solution program, using the intrinsic phasing solution method and refined against $|F^2|$ with XL [4] using least-squares minimization. Hydrogen atoms were generally placed in geometrically calculated positions and refined using a riding model. All images were rendered usingOlex2.

Herein we present the singe crystal structures of bis-alkynyl benzophenone intermediate **3** (Figure 6) and tetra-aldehyde **4** (Figure 7, top). The structure of **3** completes the list of standard TPE precursors, i.e. di(*p*-C_6H_4X) substituted benzophenones. **3** was found to crystallize in the orthorhombic space group P*ccn*, and only minor differences are observed in the comparison between **3** and **1**, amongst other halo-benzophenone compounds with regards to data such as the C-C(O)-C angle, and the plane twist angle, i.e. the degree of puckering of the phenyl rings.[5]

In contrast, **4** proved to be more of a taxing system, both to grow suitable crystals of, and to refine. Numerous attempts were made to crystallize **4** from common laboratory solvents (CH_2Cl_2, THF, CH_3OH, *i*PrOH) and in most cases **4** was found to precipitate out. Crystals of **4** were found to form upon slow evaporation from *i*PrOH but, eventually crystals suitable for analysis were grown via slow evaporation of a saturated solution of **4** from CH_2Cl_2/F_3CCO_2H. Previous structural studies involved powder X-ray diffraction experiments on covalent organic frameworks (COFs) derived from **4**.[6]

It was found that the core of **4** was quite similar to **2a**,[32] as exhibited by the overlay presented (Figure 7, bottom). However, some differences prevailed; 1) unlike **2a**, which exhibits distinct Br…Br interactions, the structure of **4** revealed no major molecular interactions of note and 2) whilst the host-guest nature of **2a** is well documented with a variety of guests, the solvent guests could not be identified in the present structure of **4** and their diffuse diffraction component removed from the data.[7]

10.1 Crystal data/analysis for 3

Crystals of **3** suitable for X-ray diffraction analysis were obtained through evaporation of a saturated solution of the compound in methanol.

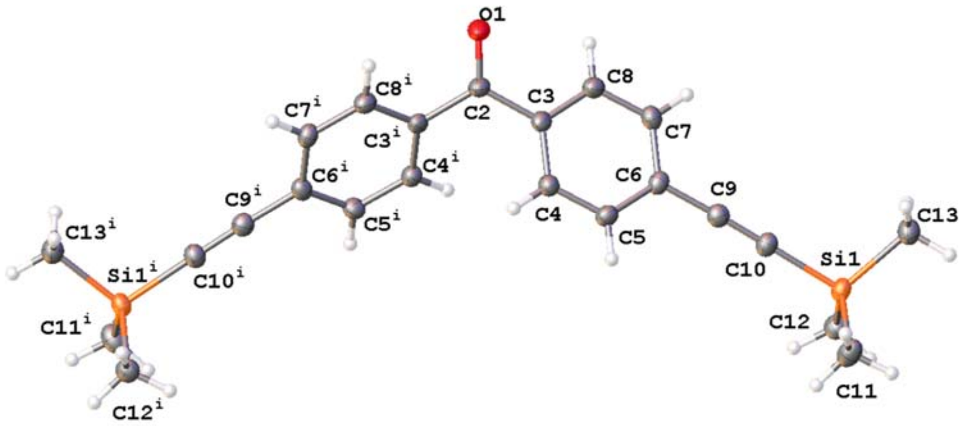

Figure S27. Symmetry generated molecular structure of **3** with atomic displacement shown at 50% probability. Symmetry transformation used to generate equivalent atoms: i = -x+1/2, -y+1/2, z.

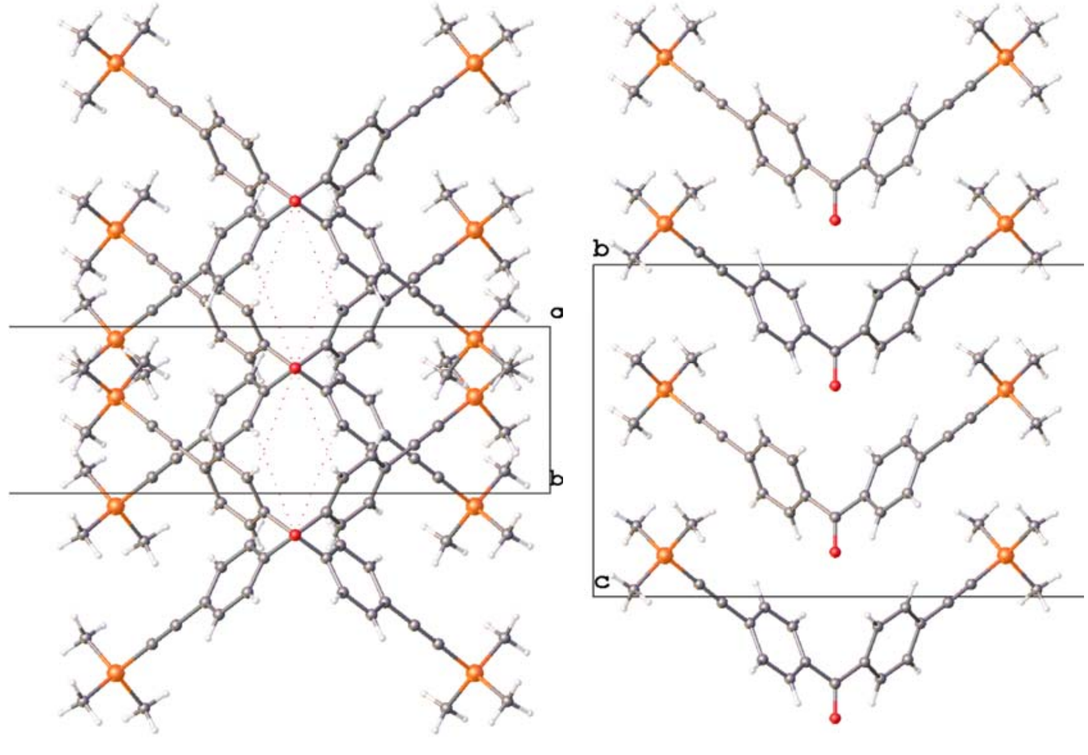

A B

Figure S28. Schematic of the packing arrangement in **3** viewed (A) normal to the a axis showing the angular stacking of column of 72.8 ° and (b) viewed normal to the b axis showing the column stacks. Dotted lines represent possible hydrogen bonding interactions.

10.2 Crystal data/analysis for 4

Crystals of **4** were grown through slow evaporation of a saturated solution of **4** in CH_2Cl_2/F_3CCO_2H.

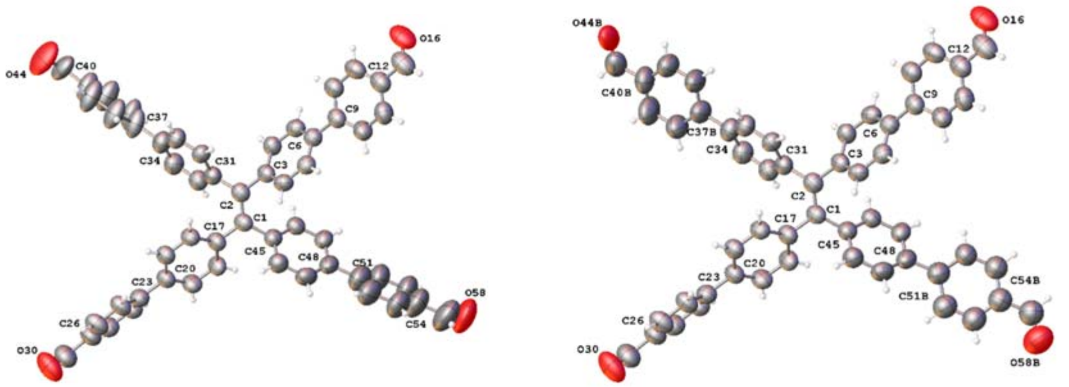

A B

Figure S29. Individual representations of each disordered moiety in **4** with (A) C37, 56% and C51, 85% occupied. Atomic displacement shown at 50% probability and all non-hydrogen atoms labelled

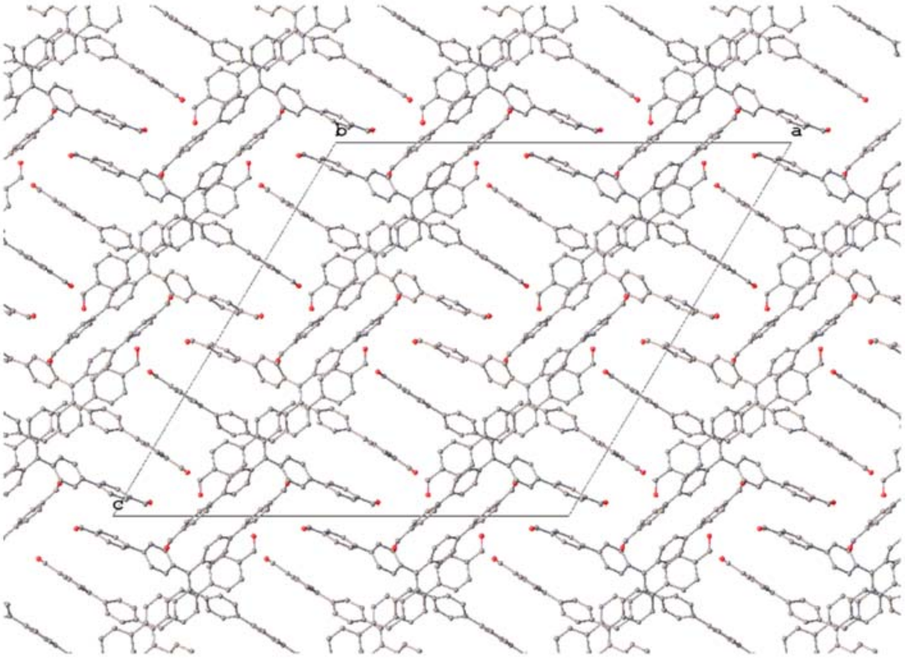

Figure S30. Schematic packing diagram of **4** with the majority occupied moiety only, viewed normal to the b-axis.

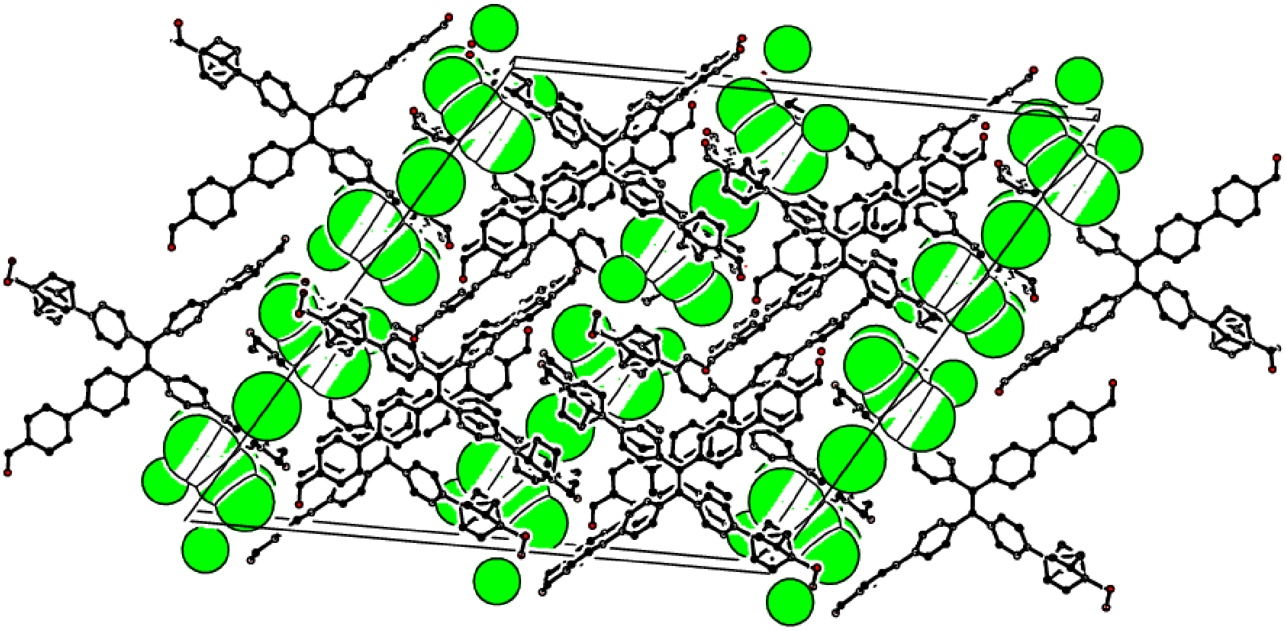

Figure S31. Solvent accessible volume in **4** calculated by PLATON. [8]

10.3 Details of XRD refinement

Table S2. Crystal data and structure refinement for **3** and **4**.

	**3**	**4**
CCDC	2063490	2063491
Internal Code	tcd1536	tcd1604_sq
Empirical Formula	$C_{23}H_{26}OSi_2$	$C_{54}H_{36}O_4$
Formula weight (g mol^{-1})	374.62	748.83
Temperature (K)	100(2)	100(2)
Crystal system	Orthorhombic	Monoclinic
Space group	Pccn	C2/c
a (Å)	33.9086(9)	35.8085(13)
b (Å)	5.5681(2)	9.0237(3)
c (Å)	11.6573(3)	34.1519(11)
α (°)	90	90
β (°)	90	120.7872(19)
γ (°)	90	90
Volume (Å^3)	2200.97(11)	9480.2(6)
Z	4	8
ρ_{calc} (g cm^{-3})	1.131	1.049
μ (mm^{-1})	1.514	0.514
F(000)	800	3136
Crystal size (mm^3)	0.31 x 0.09 x 0.04	0.36 x 0.19 x 0.06
Radiation	Cu K_{α}	Cu K_{α}
Wavelength (Å)	1.54178	1.54178
2θ (°)	2.606 to 69.836	2.873 to 58.986
Reflections collected	15541	35951
Index ranges	-39≤h≤41, -6≤k≤6, -13≤l≤12	-39≤h≤38, -10≤k≤9, -37≤l≤37
Independent Reflections	2057	6752
R_{int}	0.0423	0.0602
R_{sigma}	0.0251	0.0486
Completeness	99.9%	99.2%
Restraints	0	498
Parameters	122	585
GooF	1.069	1.213
Refinement method	Full-matrix least-squares on F^2	Full-matrix least-squares on F^2
*R_1 [I > 2σ(I)]	0.0404	0.1168
*wR_2 [I > 2σ(I)]	0.1110	0.3307
R_1 [all data]	0.0457	0.1648
wR_2 [all data]	0.1152	0.3682
Largest peak (e Å^{-3})	0.352	0.363
Deepest hole (e Å^{-3})	-.0261	-0.328

*$R_1 = \sum ||F_o| - |F_c|| / \sum |F_o|$, $wR_2 = [\sum w(F_o^2 - F_c^2)^2 / \sum w(F_o^2)^2]^{1/2}$.

Refinement details for **3**: N/A

Refinement details for **4**: Sample showed poor diffraction, resolution was limited to d = 0.9 Å. Two terminal carboxy phenyl groups were modelled as disordered in two locations using rigid groups, occupancies C37, 56%; C37B 44% and C51, 85%; C51b, 15%. Refined with restraints (DFIX, SIMU, RIGU and ISOR). It was not possible to refine the solvents in the lattice voids and their contribution to the diffraction data was removed using the SQUEEZE[5a] routine in PLATON.[5b] The solvent accessible volume (SAV) is 2025 Å^3 and there are 636 electrons found in this SAV. This is a mixture of CH_2Cl_2 and F_3CCO_2H.

References

[1] *(a) Saint, Version 8.37a., Bruker AXS, Inc., Madison, WI,* ***2013****; b) SADABS, version* ***2016****/2., Bruker AXS, Inc, Madison, WI,* ***2014****; c)* APEX3*, Version* ***2016****.9–0., Bruker AXS, Inc., Madison, WI,* ***2016****.*

[2] O. V. Dolomanov, L. J. Bourhis, R. J. Gildea, J. A. K. Howard *and* H. Puschmann, *J. Appl. Crystallogr,* **2009**, *42,* 339–341;

[3] G. M. Sheldrick, *Acta Cryst.* **2015**, *A71*, 3-8.

[4] G. M. Sheldrick, *Acta Cryst.* **2015**, *C71*, 3-8.

[5] For halogen = F: S. J. Maginn, R. J. Davey, *Acta Cryst.* **1994**, *C50*, 254-255. For halogen = Cl: V. V. Mitkevich, V. G. Lirstman, M. A. Strzhemechny, A. A. Avdeenko, V. V. Eremenko, *Acta Cryst.* **1999**, *B55*, 799-906; W. Manthey, K. Plieth, A. Singewald, *Ber. Bunsenges. Phy. Chem.* **1952**, *56*, 690-694. For halogen = Br: B. Peric, B. Kojic-Prodic, *Acta Cryst.* **2000**, *C56*, 211-212. For halogen = I: G. P. M. Van Der Velden, J. H. Noordik, *J. Cryst. Mol. Struct.* **1979**, *9*, 283-294.

[6] a) S.-Y. Jiang, S.-X. Gan, X. Zhang, H. Li, Q.-Y. Qi, F.-Z. Cui, J. Lu, X. Zhao, *J. Am. Chem. Soc.* **2019**, *141*, 14981–14986; b) A. F. M. EL-Mahdy, M. G. Mohamed, T. H. Mansoure, H.-H. Yu, T. Chen, S.-W. Kuo, *Chem. Commun.* **2019**, *55*, 14890–14893.

[7] F. M. Amombo Noa, S. A. Bourne, L. R. Nassimbeni, *Cryst. Growth Des.* **2015**, *15*, 3271–3279; K. Tanaka, D. Fujimoto, T. Oeser, H. Irngartinger, F. Toda, *Chem. Commun.* **2000**, 413–414.

[8] *(a)* P. van der Sluis, A. L. Spek, *Acta Cryst.* **1990**, *A46*, 194-201; *(b)* A. L.Spek, *J. Appl. Cryst*. **2003**, *36*, 7-11
